# Multi-touch Interaction Data Analysis System (MIDAS) for 2-D tactile display research

**DOI:** 10.3758/s13428-019-01279-1

**Published:** 2019-07-24

**Authors:** Grecia Garcia Garcia, Ronald R. Grau, Frances K. Aldrich, Peter C-H. Cheng

**Affiliations:** Department of Informatics, University of Sussex, Brighton, UK

**Keywords:** Multitouch, Tactile interaction, Visual impairment, Gesture logging and analysis, Tactile protocol analysis

## Abstract

The study of haptic perception and cognition requires data about how humans interact with tactile surfaces in the context of performing cognitive tasks. MIDAS is a set of three tools for the digital capture, coding, analysis, and interpretation of time-series, multitouch, interactive behaviors on a tactile surface. The MIDAS-logger uses the current screen technology of tablet computers to capture touches (up to ten fingers at high spatial and temporal resolution) through conventional tactile graphics that are overlaid on the screen. The MIDAS-analyser is a software program for the qualitative and quantitative analysis of MIDAS-logger touch data, which includes a fully interactive visualization of the data and a yoked display of a conventional simultaneous video recording made of the interactions. MIDAS-tactile protocol analysis (TPA) provides a scheme and a method to enable the rich coding and interpretation of tactile behaviors over multiple spatial and temporal scales. The efficacy of MIDAS was assessed against a set of criteria drawn from the successes and limitations of prior approaches to the study of tactile interactions. To demonstrate the functions of MIDAS, its three components were used to capture, analyze, code, and interpret the behavior of an experienced user and an inexperienced user of tactile graphics as they performed a shape-matching task.

Tactile reading of braille, and tactile interaction more generally, involves fingers and hands moving across the surface of a display to obtain information, such as messages encoded in a linear sequence of raised alphanumeric symbols or the spatial configuration of other raised elements. Interest in technology and techniques to capture, code and analyze tactile interactions with (nearly flat) 2-D materials, such as braille and raised line graphics, persists for several interrelated reasons. One may be interested in our basic perceptual abilities in touch, such as the appropriateness of certain tactile features for discrimination (Jehoel, McCallum, Rowell, & Ungar, 2006; McCallum, Ungar, & Jehoel, 2006), the role of tactile gestalts in perception (Gallace & Spence, 2011), or perceptual aspects reading a tactile script (Millar, 2003). Some studies have probed the perceptual differences between sighted participants who are blindfolded and people with visual impairment (e.g., Alary et al., 2009; Heller, 2002; Jehoel et al., 2006; McCallum et al., 2006). Beyond perception, there is interest in the strategies that readers employ to read braille (Bertelson, Mousty, & D’Alimonte, 1985; Breidegard et al., 2008; Hughes, McClelland, & Henare, 2014; Millar, 2003; Mousty & Bertelson, 1992; Symmons & Richardson, 2000) and whether lateralization occurs in such ability (Mousty & Bertelson, 1985). From the perspective of display technology for people with visual impairment, research has been conducted to assess the efficacy of novel devices for delivering tactile stimuli to fingers (Blazie & Cranmer, 1976; Bliss, Katcher, Rogers, & Shepard, 1970; Kaczmarek, Tyler, & Bach-y-Rita, 1997) and the back (Geldard & Russel, 1957; White, Saunders, Scadden, Bach-y-Rita, & Collins, 1970). Additionally, from a computer science angle, there is growing interest in the potential of gesture-based human-computer interaction (e.g., Wobbrock, Morris, & Wilson, 2009).

Our interest is in methods to capture, code, and analyze tactile behaviors related to the cognitive science of tactile graphics—the human information processing involved in the reading and reasoning with tactile pictures and diagrams. Although some investigations have been conducted with tactile graphics, notably Jehoel et al. (2006) and McCallum et al. (2006), as compared to studies with braille, relatively little is known about how tactile graphics are read. One potential reason for the relative rarity of studies is the greater complexity of behaviors involved in the interaction with tactile graphics as compared to reading braille. Although braille is a 2-D display at the level of letters and a whole text, reading may be characterized as a “1.5-D” process with the linear reading of lines of characters only punctuated with returns and movements down the page (Aranyanak & Reilly, 2013; Breidegard et al., 2008; Millar, 2003). So, many questions remain open. How do people read 2-D graphics by touch? How does interaction with tactile graphics vary across people with different levels of experience? How and why do their strategies differ? What constitutes the design of an effective tactile graphic? These are question that are fundamentally cognitive rather than perceptual in nature, because they involve recognition, search and comprehension processes beyond elementary detection and discrimination. We argue such questions demand data and analysis spanning multiple spatial and temporal scales. Sizes range from symbols of few millimeters width to whole displays measured in tens of centimeters. Temporal durations span ≈ 100 ms, for recognizing the presence of an object, to potentially many 100 s, for the interpretation of, or problem solving with, a complex display. Reading braille typically employs two input streams comprising one finger or a conjoined pair per hand. In contrast, interacting with tactile graphics often involves multiple fingers per hand with different fingers on the same hand potentially serving alternative functions. The example task in the next subsection illustrates the complexity of behaviors in a 2-D tactile task.

This article presents the Multi-touch Interactive Data Analysis System (MIDAS). MIDAS comprises tools and methods to address the challenges of capturing, coding and analyzing complex behaviors with 2-D tactile graphics. Our approach uses touch screen tablet computers to record the simultaneous movement of up to ten finger contacts with a tactile graphic laid over the screen. MIDAS provides tools and methods for both quantitative and qualitative analysis of that data, which span multiple spatial and temporal scales of tactile behaviors.

## Motivating example: Shape search and matching task

**Fig. 1:**
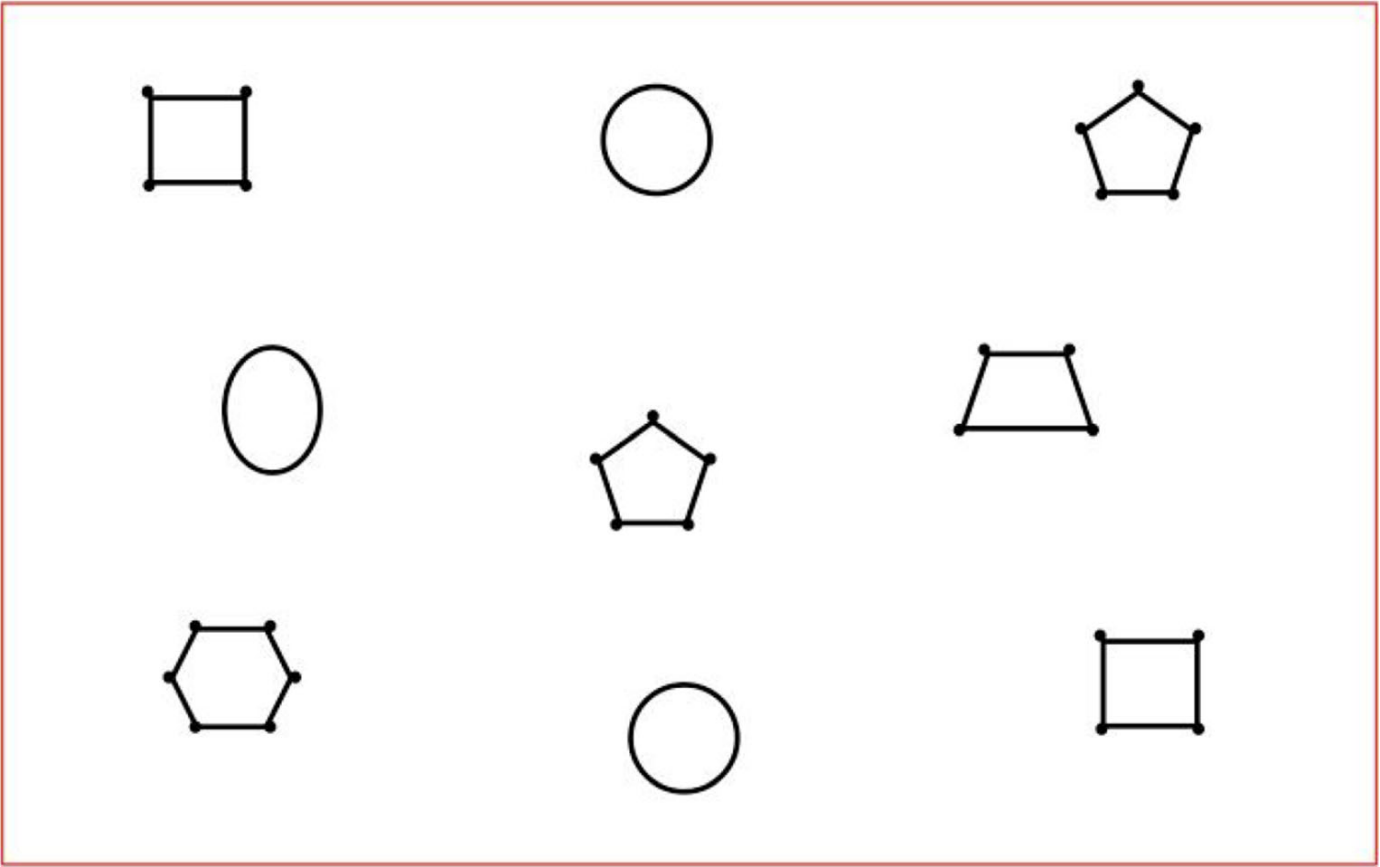
Stimulus for the shape search and match task

Figure 1 shows a 16.6 × 26.4 cm stimulus for a task that involves finding and naming similar pairs of shapes, which will serve as a running example throughout this article. (The perimeter, in red, is part of the stimulus, and its role is explained below.) To illustrate our tools and methods, we will concentrate on the trials of just two participants. Our Participant P1, *P1* (ID code E1C2), is a 16-year-old student with very low vision who reads using braille. She has experience in reading tactile material, including specific training, having attended a specialist school for children with visual impairment for 4 years. Our Participant P2, *P2* (ID code P403), is a blindfolded 22-year-old sighted adult who has little experience in reading tactile graphics by touch. Participant P1 correctly matched all the pairs and named them correctly, taking 41 s to complete the task. In contrast, Participant P2 required 3 min 11 s to complete the task, but was only successful in matching two of the three pairs.

The contrast between these participants reflects the range and complexity of tactile behaviors that must be addressed in the study of 2-D tactile interaction. (The full study focusing on novice–expert differences in reading tactile graphics will be published elsewhere.)

**Fig. 2:**
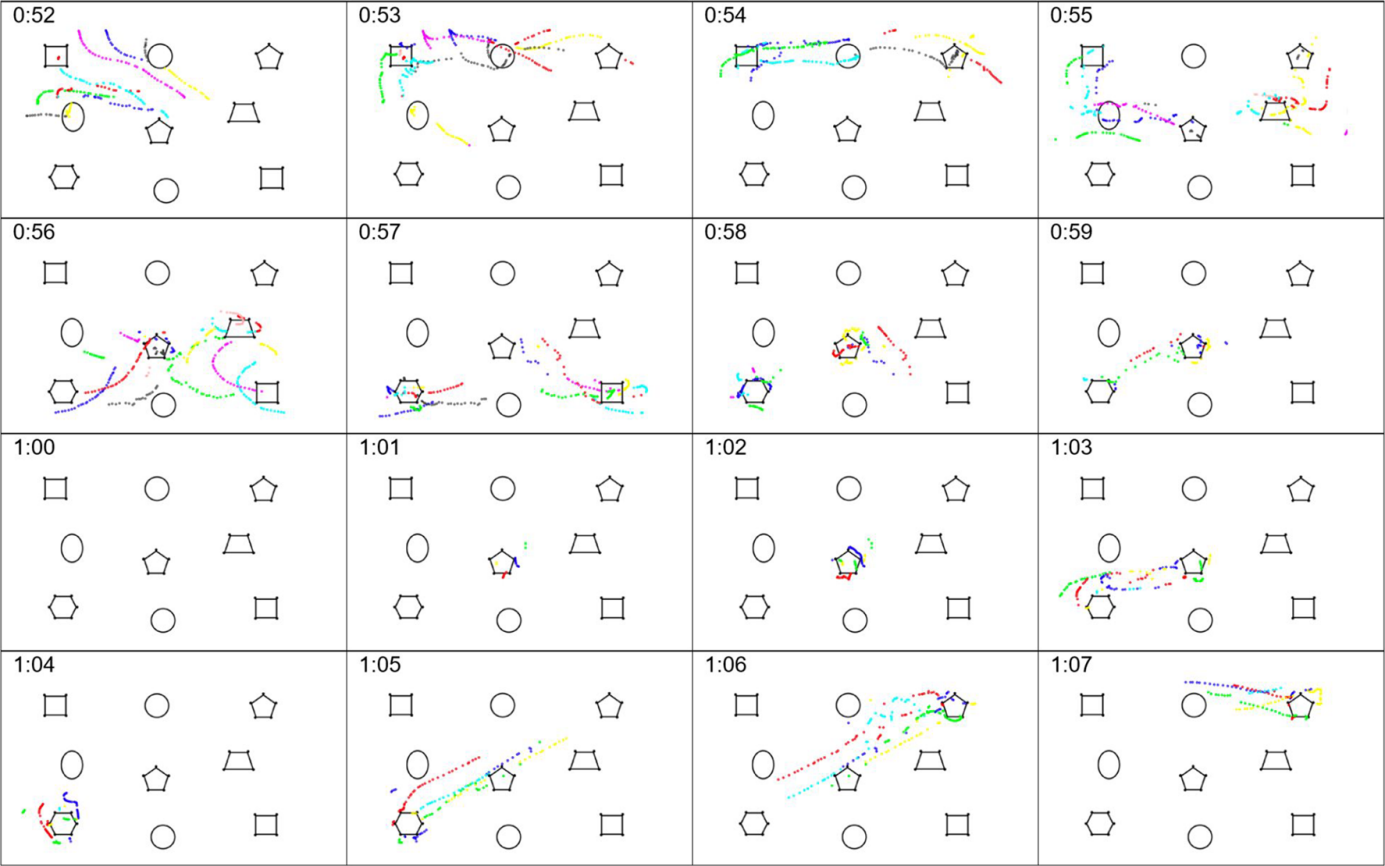
MIDAS-analyser displays of participant P1’s performance on the shape search and match task. Frame reference numbers are in minutes:seconds. Successive frames are 1,000 ms apart and have durations of 1,200 ms (first 16 s of the trial are shown)

**Fig. 3:**
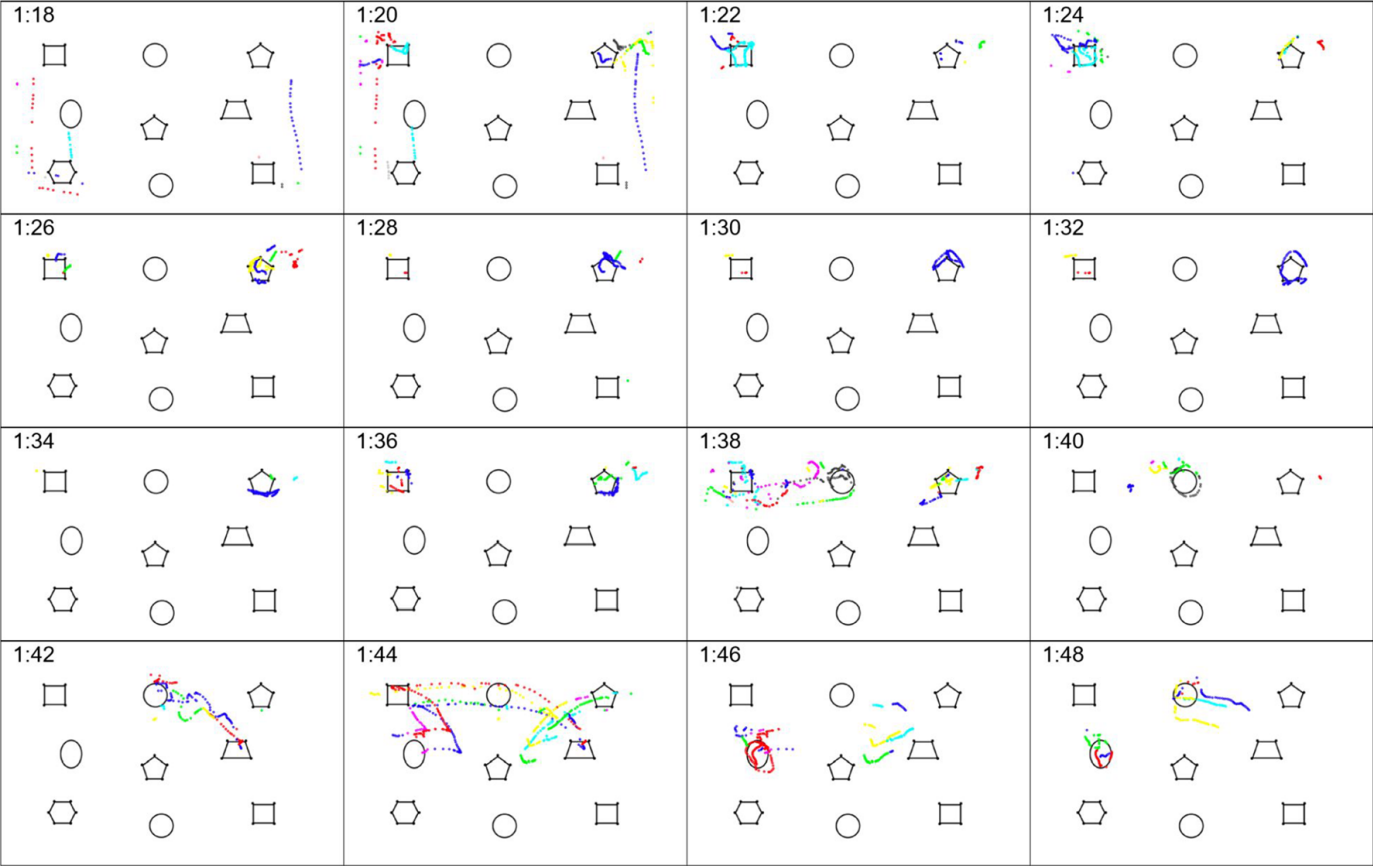
MIDAS-analyser displays of participant P2’s performance on the shape search and match task. Frame reference numbers are in minutes:seconds. Successive frames are 2,000 ms apart and have durations of 2,400 ms (first 32 s of the trial are shown)

Figures 2 and 3 are sequences of displays from the MIDAS-analyser tool for initial activity on the task of Fig. 1 by our Participants P1 and P2, respectively. Each color dot is an instance of finger contact with stimulus, sampled at an average rate of 60 Hz. A running sequence of dots indicates continuous contact of a finger with the stimulus, and different colors are separate contacts. Two distinct, disjointed runs of the same color are two separate touches and may correspond to successive movements of the same or different fingers separated by a gap; in other words, dot color represents the order of occurrence of touches, not finger identity. The 16 frames of Fig. 2 show the first 16 s of P1’s activity, with each frame displaying 1,200 ms of data, ending at the time of the frame’s time label. (P1 began touching the stimuli after about 51 s of instructions). The overlap of 200 ms between frames aids our interpretation of the flow of touches across frames. The frame duration in Fig. 3 was chosen to suit P2’s behaviors, with a span of 2 s including 2,400 ms of data and a 400-ms overlap.

The sequences convey an impression of the complexity of 2-D tactile behaviors. Interactions may involve just one digit (i.e., finger or thumb), or several simultaneously, with the use of one or both hands (e.g., Fig. 3, cf. frame 1:34 and frame 1:24, respectively). This diversity occurs locally, at the level of individual shapes, and at the level of rows and columns, too (Fig. 3, cf. 1:18 and 1:34). Movements between shapes sometimes follow the rows and columns of shapes but may sweep diagonally, too (Fig. 2, 0:54, or Fig. 3, 1:18; cf. Fig. 2, 1:06). Motions can be slow or rapid (Fig. 2, 0:58; cf. Fig. 3, 1:44), and sometimes a dense cluster of dots suggests that digits appear to be parked at a particular location, including in free space between shapes (Fig. 3, 1:40). A shape may be continuously traced with a single digit (Fig. 3, 1:32) or broken down into separate strokes with different digits (Fig. 2, 1:02). Movement trajectories are sometimes smooth but may also be curved, or even ragged (Fig. 2, 0:52; cf. 0:56).

Individual differences between participants are also apparent. P1 typically moves faster than P2: The distances covered by long movements in each frame are similar in both figures, but remember that P2’s frames are twice the duration of P1’s. P2 may dwell on a shape for many seconds with just one or two fingers, but P1 lingers much less, which suggests superior shape recognition. P1 sweeps over all the shapes initially, seemingly to gain a sense of the overall layout of the display, before dwelling on any individual shape, whereas P2 almost immediately focuses on an individual shape. P2 appears to use more than one approach to recognize a shape: In Fig. 3, frames 1:28 and 1:30, she appears to feel specific features of the shape, but in frame 1:32 she traces around its perimeter. P1 often uses two hands and multiple fingers, whereas P2 tends to rely on a few fingers and one hand at a time—perhaps she cannot integrate two streams of input. The differences between the participants suggest that diverse strategies may be in operation and are manifested as complex patterns across broad spatial and temporal scales.

Clearly, implications for the design of tools and methods to study tactile behaviors follow from the recognition of the range and complexity of these tactile behaviors. Such requirements will be considered in the next section, alongside brief reviews of current methods for capturing, coding, and analyzing interactions with 2-D tactile materials.

## Three phases of tactile experiments

To systematically review previous approaches to studying tactile interaction, and to identify potential requirements for future approaches, we divide experiments into three phases. These are (1) the capture of raw data from participants, (2) the coding of that data for measures and patterns of interest, and (3) the interpretation of the measures and patterns.

### Phase 1: Capture data

#### Existing methods

To investigate tactile interactions, we first capture behavioral data from participants as they perform a task with 2-D tactile stimuli. In the studies of Jehoel et al. (2006) and McCallum et al. (2006) preference responses, proportions of correct responses, and overall scanning times were sufficient to find perceptual effects. Symmons and Richardson (2000) video-recorded novice participants as they read simple line drawings, with a digital timer located in shot so that response latency about shape identification could be measured. However, most researchers of tactile interactions have chosen to collect information about finger and hand movements by recording the locations of fingers and the time they touch the stimulus. We may distinguish between natural and invasive methods, depending on whether the fingers and hands are unencumbered or restricted to some extent by the motion capture equipment. Natural methods use video recordings of the hands but use different approaches to record finger positions over time. Mousty and Bertelson (1985) used two video cameras to record horizontal finger movements from above and vertical movements to one side, with a digital timer in the view of the cameras. Overall durations on particular segments of their task were obtained by playing back the recording. To obtain actual motion data from these videos, Bertelson et al. (1985) manually transcribed the position of the main reading finger of each hand every 1/10th of a second, entering the grid coordinates of stimuli into an actogram; in their words, this was a “very tedious procedure.” Millar (2003), Breidegard et al. (2008), and Morash and van der Velden (2016) also filmed, but from underneath, using a transparent stimulus placed on a clear plastic sheet through which touches were made readily apparent by the whitening of the finger pads as they pressed on the surface.

Common RGB video cameras have inherent resolution and precision limitations, because of the way the data are encoded. There are two main critical issues. Firstly, even though a standard 25 frames per second capture rate is nominally sufficient (25 fps is equivalent to capturing one frame every 40 ms), the common video compression algorithms render some of the frames unusable for analysis, so substantially reducing the effective sampling rate. One solution is to use video cameras that support higher frame rates and full-frame capture, but these are expensive. The second issue is the difficulty of reliably establishing digit positions and determining precisely when touch events genuinely occur, even when multiple cameras are employed and record from different angles. When a participant uses both hands to explore a scene, one hand might visually obstruct the other, so hiding touch points from the cameras’ view.

Approaches that avoid the challenges of video recordings have been developed and provide position and timing data directly. Noblet, Ridelaire, and Sylin (1985) attached a flashing LED to the reading finger of each hand, whose positions were recorded every 40 ms using a digital camera and bespoke electronic circuitry. More recently, methods to exploit the built-in infrared technology of the Nintendo Wii video-gaming console have been developed and used to capture finger movements of readers of a refreshable braille display (Aranyanak & Reilly, 2013) and of (sighted) children using a graphical display (Garcia Garcia & Cox, 2012), with temporal resolutions of 10 ms. However, these approaches required fixing LEDs to participants’ fingers and battery packs to their wrists. Hughes, McClelland, and Henare (2014) make a similar trade-off by attaching the pen from a Wacom digitizing graphic tablet to the primary reading finger of their participants. With the stimulus placed on the tablet the approximate position of the fingertip was automatically provided with high temporal precision, but data capture is limited to one finger, which carries a cumbersome pen.

#### Requirements

We can discern various desirable data capture system requirements from the brief reviews and from our example task.

##### R1.1. Spatial and temporal resolution and accuracy

This should be high for equipment that records hand and finger movements. With modern digital technology high temporal precision is attainable (better than 1 ms) and sampling frequencies of about 20 ms (50 Hz) are easily achieved, which is sufficient for the analysis of tactile behaviors. Given the size of finger pads (≈ 10 mm) and the lower limit of perceptual thresholds of separate objects, spatial resolution of a few millimeters is sufficient for most tactile tasks (but discrimination tests may require greater precision). Spatial and temporal measures at these limits can certainly provide derived measures of sufficient precision for studying braille reading and everyday tactile graphics activities.

##### R1.2. Multifinger and hand recording

Although some previous approaches had the capability to track more than two fingers, for practical purposes they were limited to one digit per hand. Our example task shows that multiple digits must be tracked simultaneously, and ideally all five digits on both hands should be recorded. In contrast to Symmons and Richardson’s (2000) observation of single-digit reading by novices, our Participant P2 (who has little experience reading tactile graphics) used both hands and multiple fingers, simultaneously.

##### R1.3. Digit identification

The identity of the finger touching on the stimulus is potentially useful information for interpreting strategies. Invasive approaches normally maintain finger identity, which is usually obvious from context when only recordings of a single digit per hand are captured. Our observations indicated that fingers are often pressed together, sometimes overlap, and hands may be crossed, so maintaining finger identity will be both a significant technical challenge but also critical for the interpretation of interaction behaviors.

##### R1.4. Automatic recording and minimal calibration

Fortunately, digital recording methods, such as those in the studies cited above, mean that manual transcription of positions and timings is now unnecessary. However, for general convenience, it is also desirable that manual calibration should be minimized. Thus, systems that must interpolate information about touches across data samples, and that need regular recalibration during trials or data coding, are undesirable.

##### R1.5. Touch and off-surface movements

Most current methods record all finger movements, but others are superior as they differentiate motions that contact the stimulus surface and those that do not. Such information is needed to distinguish between skimming over a stimulus versus jumping between locations just above the surface: The latter is visible in video recordings but not in Figs. 2 and 3.

##### R1.6. Unencumbered hands

Methods, such as those cited above, that require the attachment of items to participants’ hands may negatively impact natural interaction behaviors, as well as requiring setup time and expense.

##### R1.7. Practical considerations

Obviously, it is desirable for a tactile data capture system to be easy and cost effective to use. Three aspects may be considered. (a) Recording apparatus that allows experiments with any form of standard tactile stimuli is desirable. For instance, methods that use transparent materials, so that pressure-induced finger pad whitening can be observed, preclude the use of opaque swell/micro-capsule paper. (b) Clearly, approaches that exploit standard off-the-shelf technology are preferable to those that need costly specialized equipment or the construction of elaborate bespoke kit. (c) It is desirable that the data capture system be quick and simple to set up and run.

### Phase 2: Code data and derive measures

#### Existing methods

This phase involves revealing patterns in, or extracting quantitative measures from, raw data to provide information about behaviors. This will include steps to segment and classify data, the calculation of derived quantities, and the identification of elementary and higher-order actions. Speeds can be derived from Euclidian distances between sampled locations divided by the time between those samples, and trigonometry used to find trajectories. For braille studies, speed may be measured in words/minute or characters/second, or some such, in addition to physical speed. Types of motion are also important to identify, including forward tracking movement during character access, regressions to previous words, and returns to the next line (e.g., Millar, 2003).

Jehoel et al. (2006) simply compute correct responses and scanning times of tactile graphic objects, which were related to particular stimulus properties of the objects. Millar (2003) manually coded videos of finger touches by writing times adjacent to braille letters on photocopies of the stimulus, one copy for each hand. Breidegard et al. (2008) developed an automatic video analysis system that finds the finger pads, whitened by the pressure of touch, in still images taken from their video recordings. The method requires the initial manual identification of digits, which their analysis program then interpolates across successive stills, but further human intervention is required when hands overlap or are entwined. Morash and van der Velden (2016) demonstrate the use of ridge detection algorithms for the identification of finger tips, which has good accuracy. Obtaining data in relation to specific hands is obviously critical if one is interested in laterality (Mousty & Bertelson, 1985), but it is also important for the analysis of reading strategies, because the hands may be acting in a coordinated manner (Aranyanak & Reilly, 2013; Bertelson et al., 1985; Breidegard et al., 2008).

With position data captured digitally measures can, obviously, be directly computed using spreadsheets, quantitative data analysis packages or by writing bespoke software. It is common practice to display positions of finger touches on an image of the stimulus to provide an overall impression of individual task performance. This requires appropriate calibration and scaling touch location to the image of the original stimulus.

Although parallels may be drawn between eye movements and tactile interactions (Breidegard et al., 2008), we consider such an analogy too potentially misleading to be useful. Eye movements are typically decomposed into fixations (relatively stationary dwells of eyes at particular locations, during which the recognition of symbols in the stimuli occur) and saccades (ballistic movements between those dwells, during which no recognition takes place). However, for tactile movements, a two-level analysis is too coarse-grained and ill-conceived. In our examples, we see actions over at least three different spatial scales: feature perception within an object; whole-object shape recognition; and interpretation of the layout of the whole display. Furthermore, movement is fundamental to haptic perception of tactile stimuli: the dynamic pattern of dots passing under the finger pads provides the signal for braille letters. Our participant with experience in reading tactile material (P1), in the example, appears able to recognize some shapes just during sweeping motions across the display without ever dwelling upon them.

#### Requirements

Various system requirements for behavior coding and derived measures are suggested by the existing methods and our example.

##### R2.1. Coding across broad spatial and temporal scales

Our observations of interactive tactile phenomena reveal: fast localized actions (e.g., a tap on an object); fast distributed actions (sweeps across a display); long localized activities (close inspection of details of an object); and long diffuse activities (searching for an object).

##### R2.2. Diverse measures

The cited studies show that many properties of tactile interaction are potentially of theoretical interest, so a system should be capable of systematically identifying them. Obvious spatial and kinematic measures include locations, velocities (speed and direction) and accelerations of touches by digits, hands, or hand parts, such as the palms (occurring, e.g., during initial quick whole hand familiarization sweeps over stimuli). Touch pressure, finger, and hand overlaps, and hovering just above the stimulus surface are other measures or behaviors that may be of interest.

##### R2.3. Coding of diverse behavioral patterns

The examples suggest that methods are required for the systematic identification of different behaviors that serve different functions in the interactions with tactile materials. Such methods should identify both elementary actions and meaningful sequences composed of those actions.

##### R2.4. Multitouch coding

Although current approaches do not provide means to deal flexibly with coding the simultaneous use of multiple fingers, our example clearly demonstrates the need for such a capability. Methods should deal with interaction strategies whose component functions are distributed over the fingers of a single hand and between two hands.

##### R2.5. Integration of multiple streams of touch data

Although direct digital capture of touch data is preferable to coding from videos, video recordings are quick and economical to obtain and can provide useful supplementary information. Thus, we can expect that methods should support the close integration of both direct digital and supplementary streams of data.

##### R2.6. Automatic coding of behavior patterns and the computation of measures (i.e., R2.1–R2.4)

This is desirable not just for saving the labor of manual coding, but also to increase the reliability and objectivity of coding by requiring the formal specification of properties and constraints that define classes of actions. Furthermore, automatic coding permits sensitivity analysis to show that the specific definitions of measures are not overfitting the data or producing putative effects through fine tuning the measures.

### Phase 3: Analysis and interpretation

#### Existing methods

To develop process-orientated explanations about tactile interactions, or simply to explore the richness of tactile reading strategies, the third phase aims to support the interpretation of derived measures and behavioral patterns in relation to the content of the stimuli and the nature of the tactile task. Naturally, standard statistical tests are used to examine relations among measures, but a particular feature of research on tactile materials is the variety of graphical presentations of data that have been devised to support its interpretation. These may be grouped according to whether patterns of movements are overlaid on an image of the stimuli, or whether measures of behaviors are interrelated abstractly apart from the stimuli.

Millar (2003) added lines to show regressions in time-stamped images of braille stimuli and plotted separate data curves of each hand in graphs showing cumulative time on task across different types of actions. Others use vertical time series displays to show braille reading strategies, by plotting horizontal hand positions on the abscissa and time as the ordinate running downward (Bertelson et al., 1985; Breidegard et al., 2008). The relation between hands is shown by the shape and extent of interleaving of the zigzag lines down the graph. Aranyanak and Reilly (2013) augmented such time-series plots with circles whose size encodes the transit time through each braille cell. Breidegard et al. (2008) went further, with color-coded lines of touch traces to identify the hands and to show reading, regressions, and returns. With appropriate scaling, lines of stimuli text may be overlaid on the graphs. Breidegard’s (2007) system additionally highlights vocalized words, by stepping each line so that each word aligns with points in a vertically oriented amplitude plot of speech intensity from an audio recording.

Hughes, McClelland, and Henare (2014) plotted graphs of velocity (*y*-axis) against position (*x*-axis) of braille readers, in which regressions are points below a zero-velocity contour. Their graphs are shaded to reveal particular phases of reading, something that can in general be done in any graph. To explore the relation between reading speed and velocity intermittency, they also plotted acceleration zero-crossings against mean finger velocities.

Bertelson et al. (1985) showed the impact of different types of braille text reading strategy using shaded segmented bars to reveal the extent to which hands are reading alone or simultaneously along individual lines of stimuli. To examine laterality in braille reading Mousty and Bertelson (1985) plotted speed–speed Cartesian graphs with different hand combinations on each axis and overlaid the plot with contours radiating from the origin to degree of superiority of, for example, one versus two handed reading.

Breidegard (2007) augmented his finger-tracking system (Breidegard et al., 2008) with tools to support the semi-automatic alignment of participant verbalization with the stimuli text at the syllable level. Rather than using speech recognition, which was not reliable (especially for Swedish), points of high-amplitude speech in the audio recording were identified by computer, and a markup tool was provided for users to tag positions in the stimulus text where the vocalization of syllables was likely to be loud. So, when the audio was replayed, the computer would highlight on a screen image of the stimulus what words were being spoken. After any mismatches between the audio recording and display were corrected, by the user editing the tags, the system was then able to simultaneously show the words being read by finger and the words being spoken aloud.

#### Requirements

The types of analysis appropriate to a study will naturally depend on the theoretical perspective held. However, for our purposes it was nevertheless useful to identify classes of analysis in terms of alternative or general perspectives.

##### R3.1. Finger and hand focus

This perspective simply interrelates measures associated with the digits or hands, without particular concern for the structure of the stimuli. For instance, how does the number of digits used, or how does the speed of movement, vary with experience? Standard statistical tests across groups of participants, on measures of central tendency and dispersion of the variables mentioned in Phase 2, will be typical here.

##### R3.2. Stimulus focus

This analysis focuses on the patterns, types, and amounts of activity in different areas of interest of the stimuli, without particular concern for the sources of the contacts. For instance, what is the distribution of touches across different areas of interest in the stimuli? The focus could be, for example, on individual shapes or on the rows versus columns in Fig. 1.

##### R3.3. Measures in context

By combining the previous two perspectives, this requirement recognizes the importance of relating patterns and measures of finger activity to the detailed structure of the stimulus. For instance, the value of understanding finger movements in relation to stimuli is apparent from the number of approaches that overlay trajectories onto images of stimuli or that relate measures to areas of interest within stimuli, as can be seen in several of the methods cited above.

##### R3.4. Integrating nontouch data

Breidegard’s (2007) combination of users’ verbalizations with touch data suggests the wider possibility of triangulating touch data with other behavioral or physiological data. For example, one might wish to capture eye movements of people with partial sight as they read tactile graphics, or assess sighted users on a touch screen device that has rich localized haptic feedback.

### Summary

From our review of previous approaches and our example in Figs. 1, 2, and 3, we identified 17 requirements for systems for the experimental study of tactile interaction. The requirements provide a basis for comparing alternative approaches. Together, they may serve as a guide to those who are designing systems for the investigation of tactile behaviors and also, more widely, as guidance for researchers who are conducting such studies.

The remainder of this article presents the MIDAS tools and methods for the capture, coding, and analysis of interactions with 2-D tactile materials. MIDAS-logger (https://github.com/rrgrau/midasLogger) is an Android software app for recording interactions with tactile graphics overlaid on a tablet computer screen. MIDAS-analyser (https://github.com/rrgrau/midasAnalyser) is a desktop program for the interactive visualization and analysis of MIDAS-logger data (Figs. 2 and 3 provide examples of its output). MIDAS-TPA (for “tactile protocol analysis”) is a method for coding tactile interactions at multiple levels of spatial and temporal granularity. A running case study is provided by comparing the performance of two participants (one experienced and one inexperienced in the use of tactile materials) on the shape search and matching task.

### MIDAS-logger

A key innovation of MIDAS-logger was to exploit the built-in capacitance-based touch detection capabilities of tablet computers to record touches, by placing tactile stimuli directly onto the screen of a tablet computer. This section describes the MIDAS-logger software and illustrates its use in the experimental trials with our participants.

#### Tablet software

MIDAS-logger is an Android application for the recording and processing of multi-touch inputs of up to ten digits simultaneously, but not the identity of the fingers. The device used in our initial experiments was a Samsung Galaxy Note PRO tablet. This has a 12.2-in. (296 × 204 mm) WQXGA LCD touch screen with a resolution of 2,560 × 1,600 pixels. The size proved sufficient for use with tactile graphics paper of standard A4 size. Later, a Samsung Galaxy View tablet computer was adopted that provided a larger screen size of 18.4-in. (452 × 276 mm) at full-HD pixel resolution, increasing the touch-sensitive area by about a half horizontally and a third vertically. Due to their standard screen refresh rate, both devices achieve an average sampling rate of 60 Hz, which allowed the capture of individual touch events that were on average less than 17 ms apart. Both devices used the Android operating system, version 5.1. The adoption of tablet technology addresses many of the data capture requirements given above: spatial and temporal resolution and accuracy (R1.1); multidigit recording (R1.2); unencumbered hands (R1.6); and standard technology (R1.7b). However, unique finger identification is not supported (R1.3).

At the heart of the implementation is an extended SurfaceView class that serves three main functions: First, the detection and processing of different touch events to be recorded; second, the output of touch visualizations if enabled in the program settings; and third, ensuring reliable data collection by detecting and alleviating any situations in which the touch sensor might stop recording data temporarily. This can happen with the kinds of devices used whenever a large portion of the capacitive screen is touched simultaneously, so, for instance, if the palm of a hand is laid flat on top of the screen surface and then moved across.

**Fig. 4:**
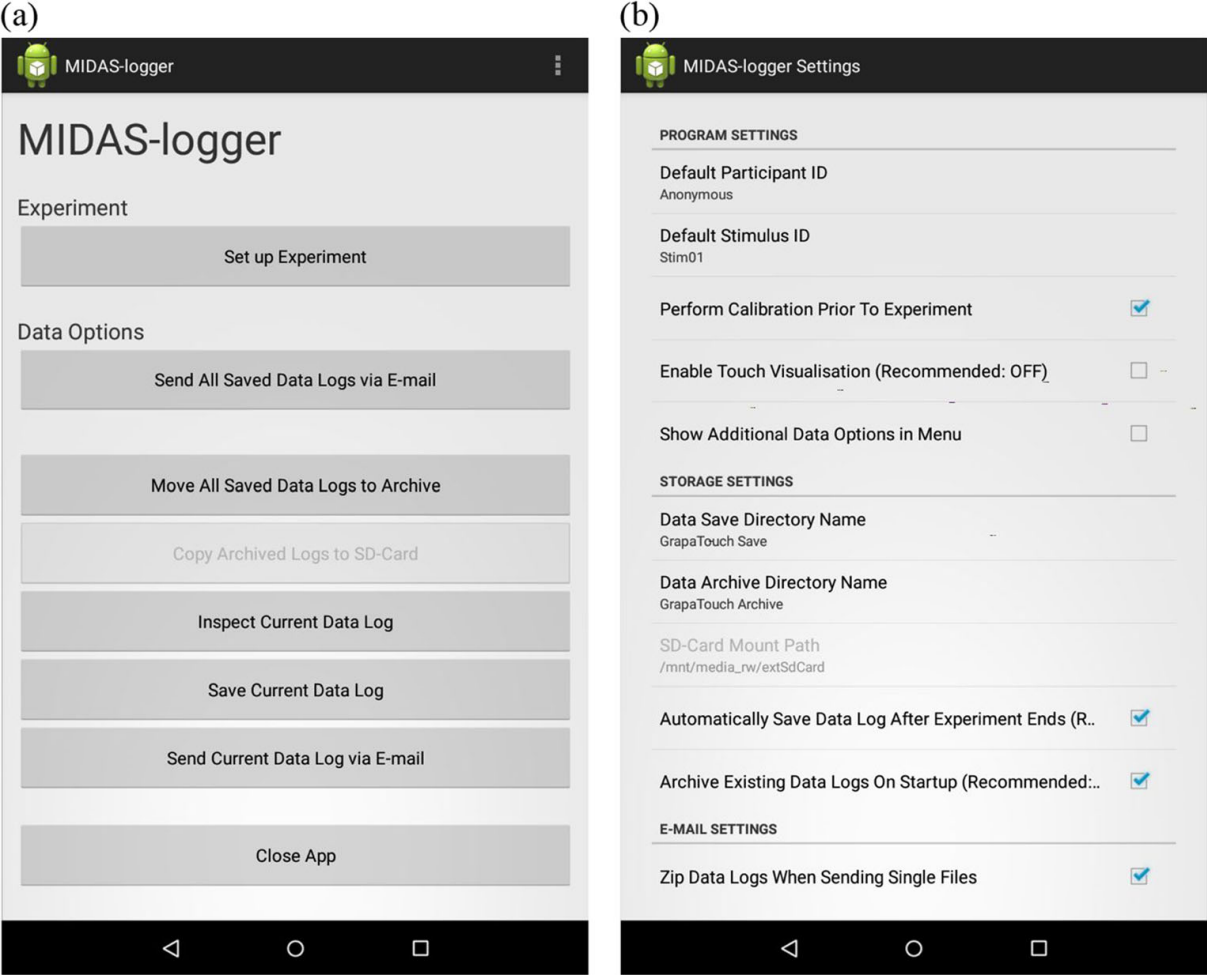
(a) MIDAS-logger home screen with advanced options enabled (left); (b) MIDAS-logger application settings (right)

MIDAS-logger supports multitrial experiments that store individual sessions in appropriately named files. After an experiment is finished, the data can be exported via e-mail. The interface is simple to use as any multistep processes involved are laid out in a wizard-like sequence. Upon startup, the basic view requires just three buttons for these operations: set up and run an experiment; export the data afterwards; and close the application. A user may enable additional features on the home screen—for instance, to inspect recorded data logs directly on the device, or to access additional archiving and data export options (Fig. 4a).

The application configuration settings (Fig. 4b) allow the specification of diverse default entries to be used in the experiment setup. Other options relate to local data and archiving directories, calibration and visualization settings, and automatic saving and archiving of the recorded data, as well as to data compression and compilation settings, for those experiments that involve multiple trials recorded in sequence.

MIDAS-logger works on tablet computers with different resolutions and screen sizes. A calibration method is provided, for precise alignment of touch coordinates on the device to the features in the tactile stimulus, because in practice there may be small deviations (1–2 mm) from the target location when affixing the paper onto the active surface of the device. Calibration also informs the coordinate system translation that is carried out before analysis, which is needed to scale the resolution of the tablet screen to the resolution of the visualization application window and make sure data points are visualized in the correct location.

**Fig. 5:**
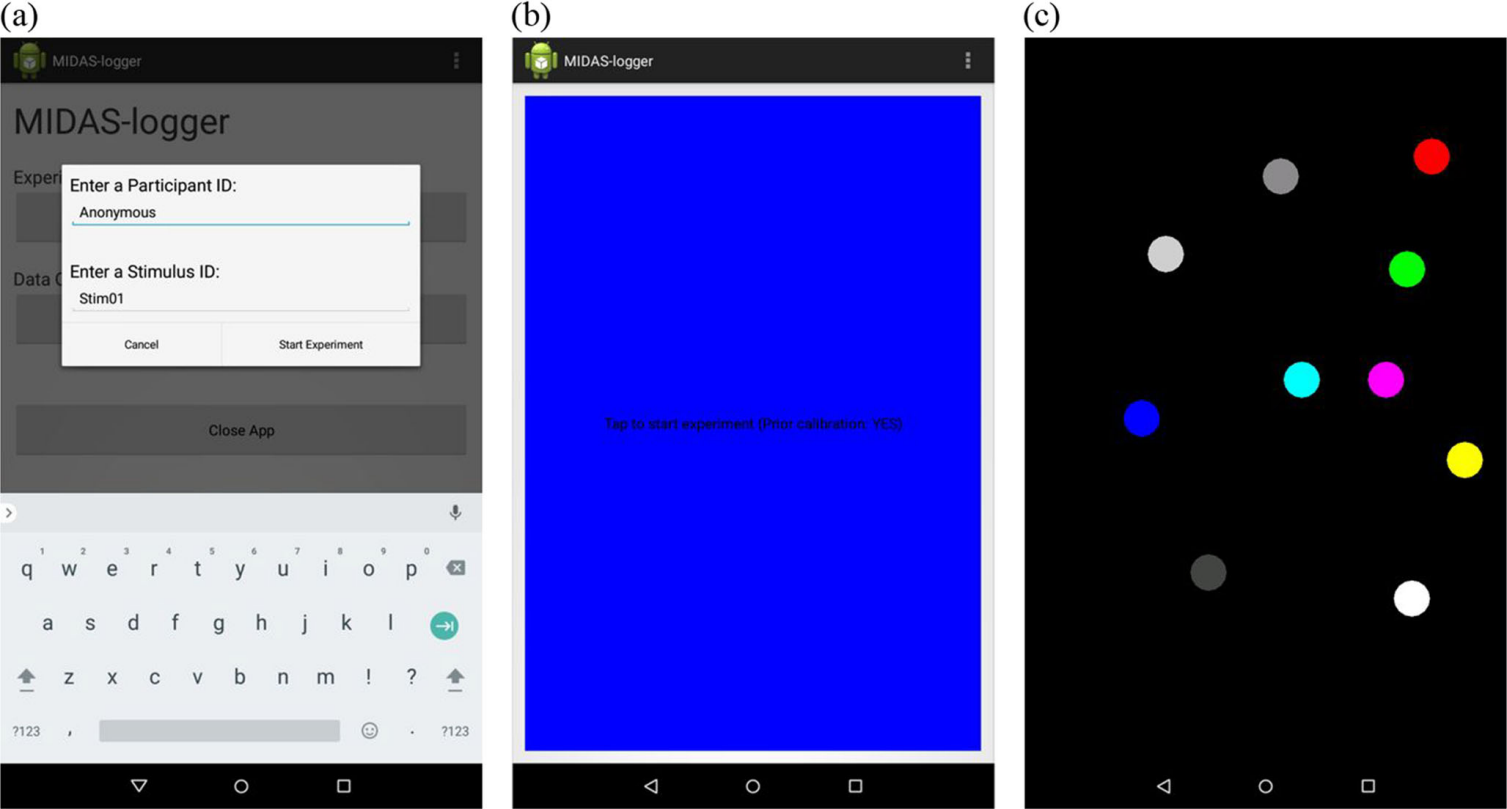
(a) Experiment setup (left); (b) calibration (middle); and (c) touch visualization (right)

Setting up an experiment with MIDAS-logger is straightforward (satisfying R1.4, minimal calibration), so only a few steps, each simple, are needed to run the software (R1.7c, simple to operate). First, the experimenter enters identifiers for the participant and the tactile stimulus used during the session (Fig. 5a). When the “Perform Calibration Prior to Experiment” option is enabled in the settings, the screen will turn blue, signaling the experimenter that the device is ready for calibration (Fig. 5b). The tactile paper with the stimulus is placed over the screen at this point. The blue light emitted by the screen, which can be seen through the tactile paper, signals to the experimenter initiation of the calibration procedure. This involves touching four marks in the far corners of the stimulus, in sequence and a specific order. MIDAS-logger emits audio feedback at key steps to ensure correct completion of the calibration process—short beeps indicate corner calibrations points have been registered successfully and a longer low-frequency tone signals that the calibration process is finished. The calibration data are stored at the beginning of the log file in a clearly distinguishable format.

Following the calibration procedure, multitouch input can begin. The screen remains black and no audio feedback is provided during normal data capture. A useful feature for testing and training, when the tablet processor load is not of particular concern, is the dynamic display of touched points as distinct colored circles (Fig. 5c) (“Enable Touch Visualisation” option). Furthermore, MIDAS-logger issues a short audio warning in the event that the tablet screen touch sensor gets overloaded. This may happen if the area touched is so large that individual touch points cannot be differentiated and properly localized, such as when participants rest their palms on the screen or if several digits of the same hand are gathered together. To unblock the sensor, participants are trained to lift their hands briefly, as will be described in the Method section.

To end an experiment, the experimenter removes the stimulus and clicks the “Back” button on the device and a long audio tone confirms the end of the trial.

#### Raw data format

The data captured are stored in a plain-text file and comprise a list of comma-separated quintuples, each describing a single touch event/state:
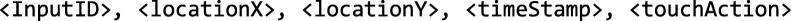


The first element is the InputID that is associated with a particular digit touching the surface, and its value can be an integer between 0 and 9. The IDs are assigned automatically and in sequence by the software, whenever any number of digits are touching the tactile stimulus. An input ID for a digit is maintained as long as the digit remains in continuous contact with the surface. The position of each touch event is recorded in screen pixel coordinates (locationX, locationY) along with a system timeStamp (in milliseconds). The last code indicates the type of touchAction recorded (Digit UP = 0, Digit DOWN = 1, Digit MOVE = 2, SENSOR OVERLOAD = – 1, CALIBRATION = *).

Given the achievable sampling rate (ca. 60 Hz), a typical 5-min experiment could theoretically generate a data set of just under 18,000 recorded touch states per digit (180,000 in total for ten digits used simultaneously). The actual amount tends to be about half this, as not all digits are used at the same time and to the same extent. Furthermore, there are natural short periods of inactivity between different touch actions and also longer pauses during an experiment (when the participant is listening to instructions, for example).

#### Method

To illustrate the use of MIDAS-logger, we describe the method used in our shape-matching task.

##### Equipment

The stimulus in Fig. 1 was printed on Zychem swell paper using a high-speed laser printer, so that the smooth, low-resistance (friction) feel of the paper was retained. The paper was run through a Zyfuse Heater (www.zychem-ltd.co.uk/zyfuse-heater/4578411322) on a medium setting two or three times to obtain a uniform raised line height. The lines were 1 mm (3 points) wide and were raised by approximately 0.5 mm.

**Fig. 6:**
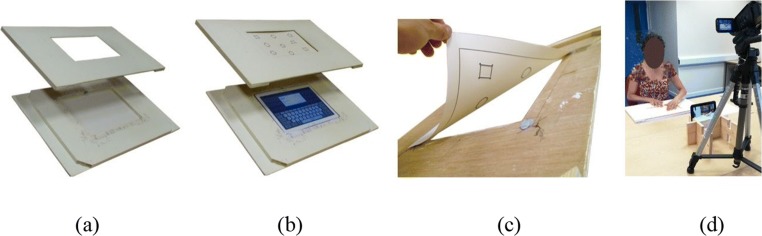
Equipment and experimental setup: (a) base and cover of wooden frame; (b) tablet computer and tactile stimulus in place; (c) attachment of stimulus to underside of cover; and (d) participant with tablet and arrangement of video cameras

For the Galaxy Note Pro tablet, we made a two-piece painted wooden frame that holds the tablet and stimulus (Fig. 6a). The tablet sits snugly in a hole in the bottom frame, with the tablet screen flush with the frame’s top surface (Fig. 6b). The stimulus is fixed to the bottom side of the upper frame (with small blobs of reusable self-adhesive putty, Blu-Tack), so that the red perimeter of the stimulus aligns with the inner edge of the opening (Fig. 6c). The size of the opening slightly overlaps the active area of the tablet screen so that the tablet pull-down/up menus cannot be activated accidentally. The frame is placed in front of the seated participant, on a table of suitable height, and is held in place to prevent sliding (with Blu-Tack again). Figure 6d shows the arrangement, with additional video recorders capturing front, side, or elevated views, as desired. Participants were asked to push up their sleeves and remove bracelets. (For the Galaxy View tablet, a single-piece, wood-and-cardboard frame was made as a simple overlay.) These basic components and simple procedures satisfy requirements R1.7a and R1.7c: Other types of tactile material can be used provided that they are thin and the elevation of the surface is limited, and only rudimentary workshop skills are needed to make the frames.

##### Procedure

Each participant was familiarized with the setup. Participants were specifically trained to generate touch sensor overloads (see above) and practiced lifting their hands from the stimulus in response to the audible signal. Participants quickly learned to stop contact with the surface briefly and to resume where they had left off, so that before the experimental trials they were responding rapidly without prompting from the experimenter. The frequency of occurrence of such overload events depends upon the particular user and the specific tablet model used, but they are generally quite rare throughout.

The experimenter performed the calibration procedure prior to each trial. The task instructions to the participants were:

The display that I put in front of you has 9 shapes. For this task, I would like you to go through each shape and see if you can find an exact match. There are 3 matching pairs in this display only. That means that for each pair there is an additional shape, which is similar, but it is not the same as the pair. So, once you have found the 3 pairs then that means that you have finished. You will have 5 minutes to do this task—are you ready? Remember to speak aloud whatever is on your mind. You can start now.

For the full shape-matching task (calibration, task, and debrief), the session with Participant P1, who had experience using tactile materials, generated 9,696 lines of touch data in 2 min 55 s; the data from Participant P2, who did not have experience using tactile materials, generated 13,760 lines of touch data in 4 min 49 s.

### MIDAS-analyser

MIDAS-analyser supports the coding and derivation of tactile behaviors from the raw data collected by MIDAS-logger and integrates those data with supplementary video recordings made of a trail. The images in Figs. 2 and 3 are screen snapshots from the touch visualizer of MIDAS-analyser.

#### Data processing and alignment

The primary functions of MIDAS-analyser are (1) the automatic processing and transformation of touch data into various formats; (2) combining different data sources with the captured touch data; and (3) visual reproduction, controlled replay, and segmentation of all data collected. (The requirements, in the second and third sets, that particular design features satisfy are noted in parentheses.)

The data processing creates a time-ordered, synchronized representation of the tablet touch data and the video recordings. A key technical challenge is the coordination of the data streams (R2.5). First is the requirement to align the data streams from capture devices with different native sampling rates: for instance, a common video camera would sample at up to 25–30 Hz, or a SAMSUNG tablet computer, at 60 Hz. Second, the tactile stimulus dimensions and location are scaled to match the coordinate systems of the recording devices. Third, control of the audio/video and digit visualization replay feature must be synchronized.

Synchronization is required because the video stream is continuous, whereas the touch data are captured only when touch events actually occur (no event, no data), so routines have been developed to coordinate each separate sequence of touch events with its own offset with video frames, taking into account the differing touch sampling and video frame rates.

**Fig. 7:**
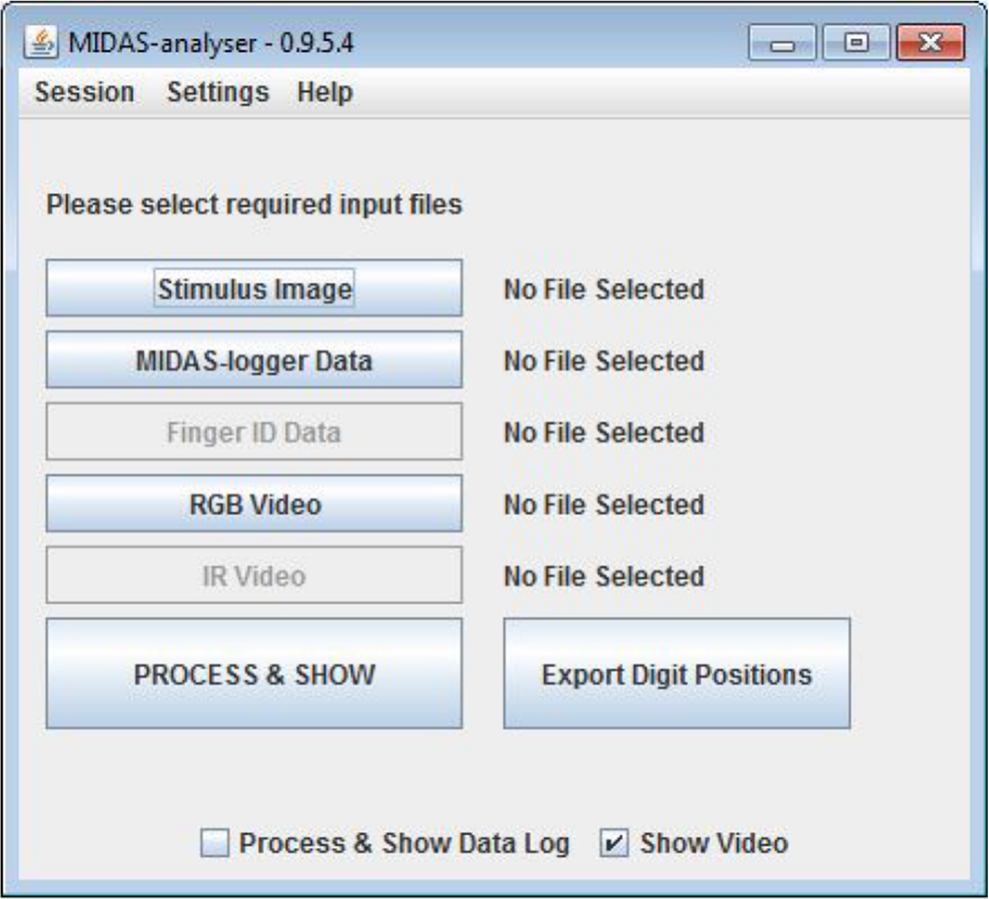
Data analysis tool startup window

MIDAS-analyser is implemented as a Java program, to enable cross-platform use. On startup, the home screen prompts the user for basic configuration settings and the files required for analysis (Fig. 7). Currently, MIDAS-analyser (version 0.9.5.4) takes three inputs: (a) a digital image of the tactile stimulus; (b) a touch data file output from MIDAS-logger; and (c) an MPEG4-encoded RGB video file. In the settings menu, the screen resolution of the touch data capture device, as well as that of the visualization window, can be entered before processing any input data. The original image file, from which the physical stimulus was produced, is used by MIDAS-analyser. These stimulus images are prepared with a predefined touch capture area, indicated by a red frame (see, e.g., Fig. 1), which the system uses to automatically map the actual position of the stimulus paper on the tablet surface to the logged touch data by calculating the offsets and necessary scaling using the calibration positions registered by the experimenter, which are recorded at the beginning of the MIDAS-logger files. The experimenter must manually trim the video using a video editing application, so that the start and end of the recording corresponds to the touch data in the log. The audio signals given by MIDAS-logger at the beginning and completion of each experimental trial are used like a kind of digital clapper board and so, help facilitate this task.

On clicking “PROCESS & SHOW” MIDAS-analyser parses the touch data file and performs the necessary conversion and scaling processes required for the correct visualization of the data points and their synchronization with the video recording. This creates a data timeline with a millisecond-precision temporal resolution against which all touch events and video frames are cross referenced. Then the touch visualization window appears.

#### Touch visualization

**Fig. 8:**
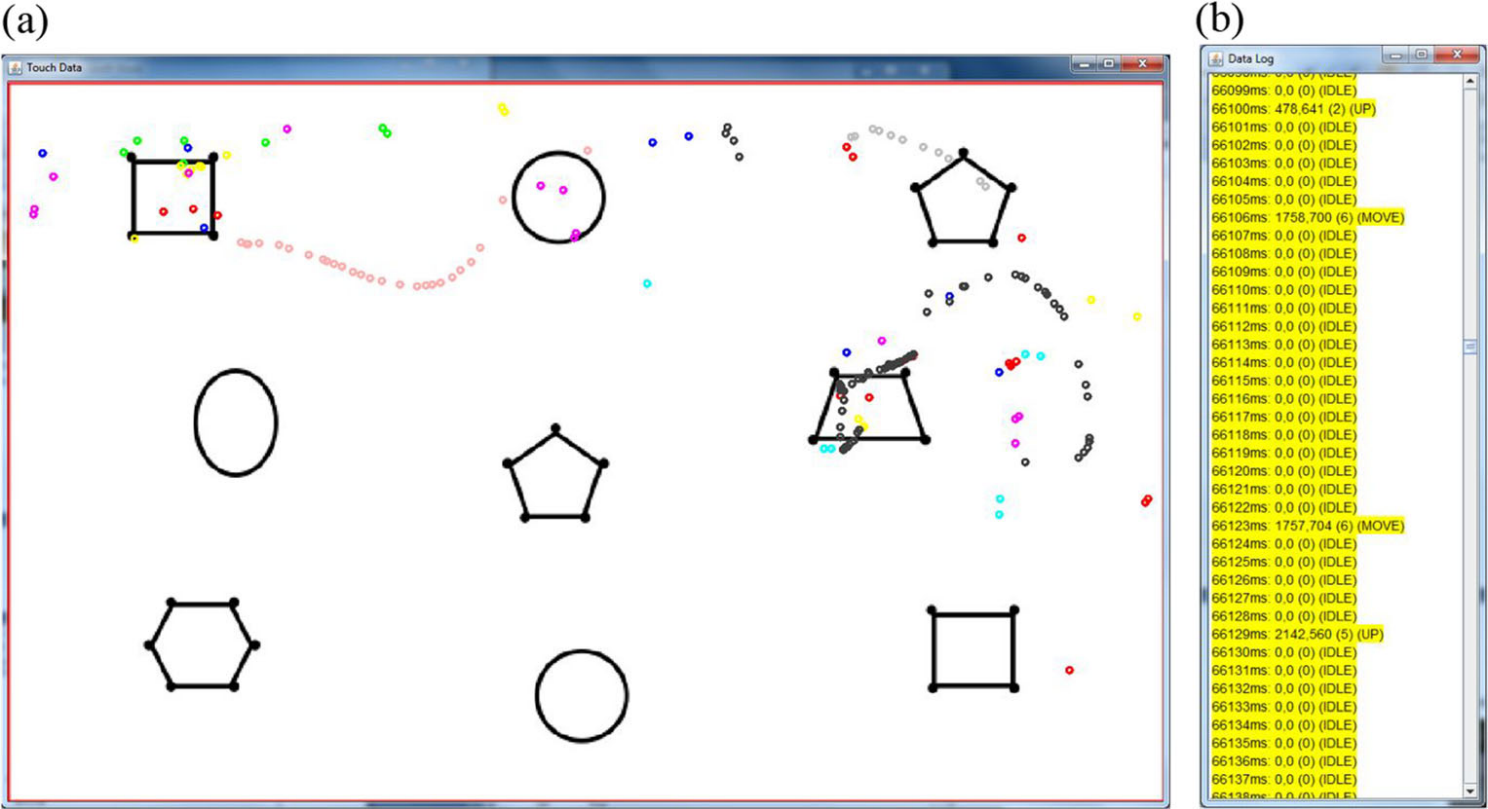
(a) Visualization window showing touch trails (left); (b) millisecond data log (right)

**Fig. 9:**
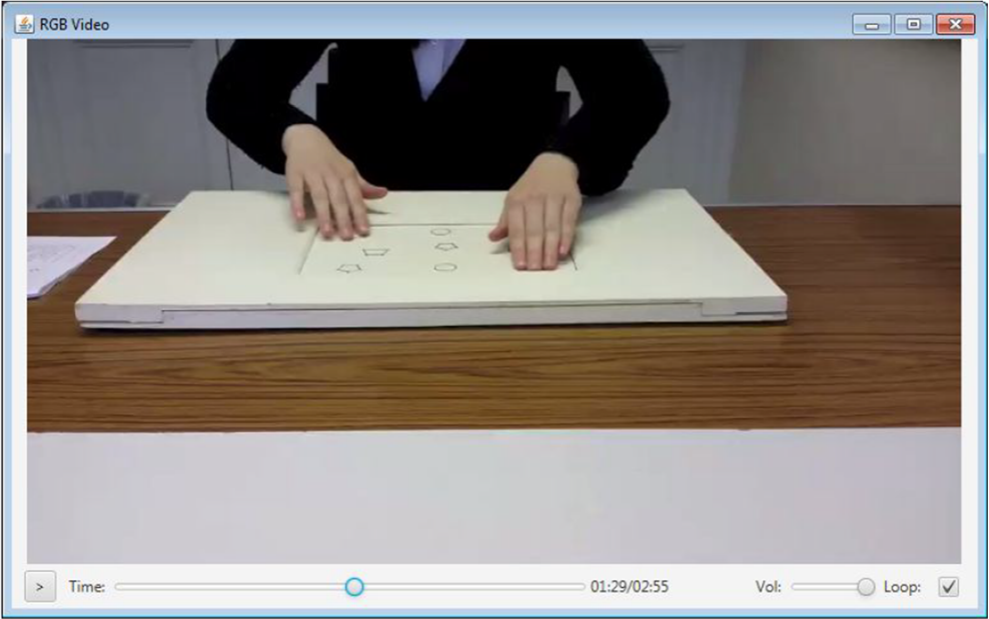
Video display window

MIDAS-analyser’s visualization window is shown in Fig. 8a (R3.1, R3.2, and R3.3). The touch data visualization is superimposed on the stimulus image for best orientation. Each distinct touch (input ID) is shown in a different color, which is not specifically associated with a particular finger (MIDAS-logger does not identify fingers): Two successive trails with the same ID (color) separated by a gap may represent different fingers. The millisecond data log, Fig. 8b, provides a time-ordered list of individual touch events at highest temporal resolution (each data row represents 1 ms on the timeline), with labels as described above (e.g., UP, DOWN, MOVE), and additionally IDLE for absence of touch events. Figure 9 shows the video display window (R3.4 for audio and video).

**Fig. 10:**
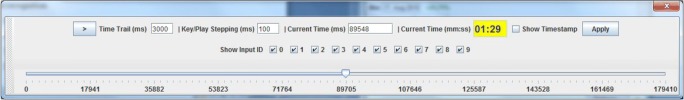
Control panel

The control panel, Fig. 10, supports navigation and replay of the data visualizations across all windows displayed. The play “**>**” button starts automatic replay of the touch data visualization in synchronization with the video recording, which would typically run at 25 frames per second. Since the video has a lower sampling rate (40 Hz) than that used for recording touch inputs (60 Hz), it is important to point out that it is the data log that is driving the visualization and not the video recording. The position in the video timeline will be interpolated relative to the data log timestamp, which is more than sufficient for visual inspection at this high level of temporal precision. The speed of replay is set by the “Key/Play Stepping” value, with a default of 100 ms/s (10% of real time). This value is also the step size for manual advances/rewinds, when the keyboard arrow keys are used to control the replay. The “Time Trail” value sets the offset (in milliseconds) of historic touch events displayed. The step value and trail length can be judiciously selected to aid visual analysis: For instance, a longer time in Fig. 3 suits Participant P2’s data better than the shorter time for Participant P1 in Fig. 2.

A user may start and stop the replay at any time, enter a specific time (in milliseconds) to jump directly to that position, or just use a slider bar for the same effect. Manually dragging the slider bar allows easy scrolling back and forth through a trail at different rates, enabling close inspections at a fine scale. Each data point can also be displayed with time stamp annotations shown, if needed. The row of checkboxes underneath the slider bar allows full control over which input IDs appear in the visualization. The yellow box indicates the current time stamp displayed in minutes and seconds, for convenience.

#### Data by digit positions

MIDAS-analyser provides data export in a format that is particularly suited for analysis using spreadsheets or analysis packages (“Digit positions file” in the home screen). The raw touch data are parsed and reformatted to create a file that contains information about the state of all ten (potential) touch inputs ordered by timeStamp, in this format:
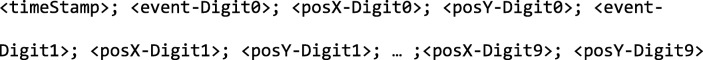


where <event-Digit <x>> is an integer indicating the event recorded at the indicated time for the respective digit. The event types are: 0:UP, 1:DOWN, 2:MOVE, 3:STAT, – 1:ERROR. STAT stands for stationary. Errors correspond to the sensor overload condition. No entry between semicolons indicates no event for that input at the time. The <posX/Y-Digit <x>> are the screen coordinates where the event took place. The advantage of representing the original data in this format is that the state of all inputs is made explicit for every time stamp (including digits not touching the surface) at each recorded time.

#### Task results from log data

To illustrate the use of MIDAS-analyser, we describe the results obtained from the shape identification trial with our two participants (illustrating requirements R2.1, R2.2, and R2.4). The first 16 s of Participant P1’s (P1) touch behaviors are shown in Fig. 2, and the first 32 s of Participant P2’s (P2) are shown in Fig. 3. The extracts shown above are quite representative of the rest of the two participants’ overall performance over the following 41 s or 3 min 11 s of their respective trials. Overall, by inspection, P1 appears to be making more simultaneous touches and moving faster.

**Fig. 11:**
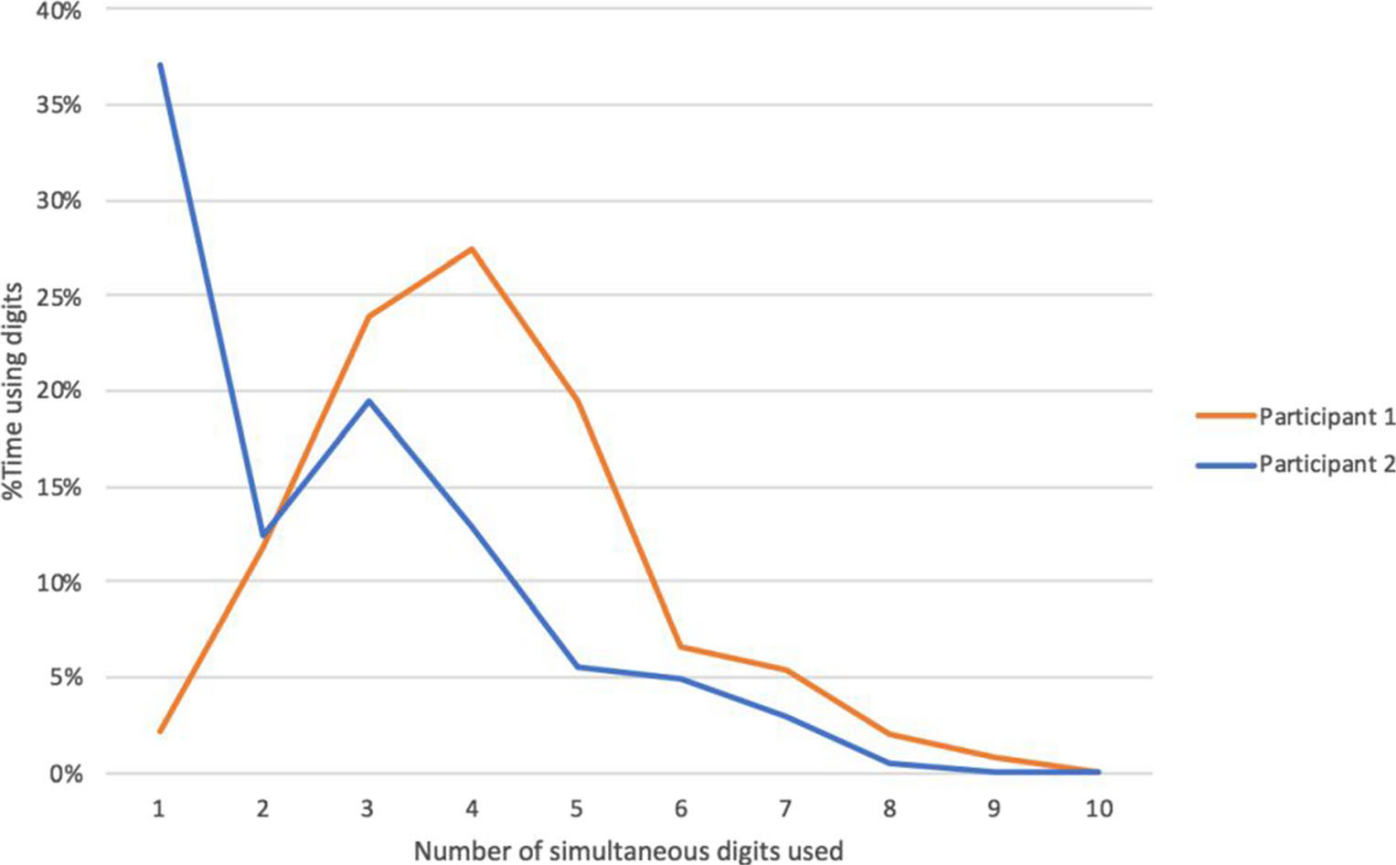
Distributions of the numbers of digits in use simultaneously

To quantify these apparent differences, the digit positions data from MIDAS-analyser were examined in a spreadsheet. To assess the extent of simultaneous touches, the proportions of time when one to ten digits were in use (stationary or moving) were computed from the logs. Figure 11 shows the distribution. The modal number of digits in use simultaneously is four for P1 and one for P2. P1 typically uses between three and five digits relatively equally, and occasionally six and seven. In contrast, P2 is heavily reliant on just one digit, sometimes uses two to four digits, and less frequently five to seven. Use of eight or more digits is rare for both participants.

**Fig. 12:**
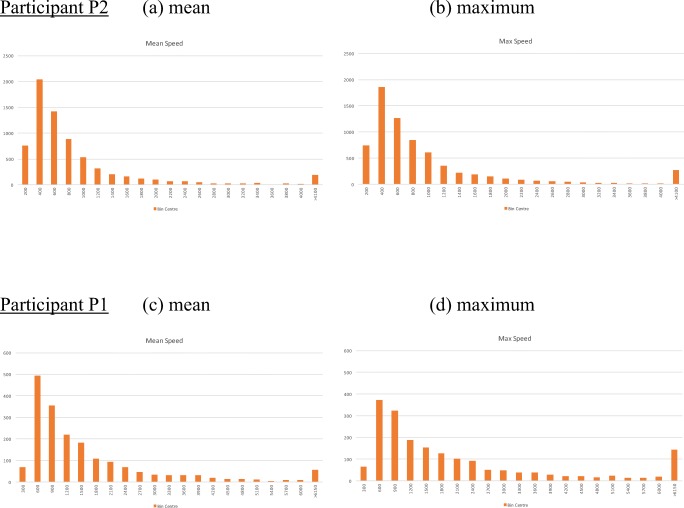
Distributions of the mean and maximum speeds of touch movements for the two participants. Histogram bin size: Participant P2 = 200 pixel/s; Participant P1 = 300 pixel/s. The *y*-axis is the number of records

To evaluate the differences in speed of touches, the distributions of the mean and maximum speeds were computed. To calculate speed of a touch movement at a given time, the Euclidian distance between a touch in a record and the immediately preceding record was divided by the time between the two records. The mean speed is the mean of the speeds of all the touches in one record and the maximum speed is the greatest speed in the record: For example, when there is just one digit, the measures are equal, and when one digit is stationary and another moving, the mean is half the maximum speed. Figure 12 shows the distributions of the mean and maximum speeds for the two participants, as overall proportions of the numbers of records. Although the shape of the participants’ distributions are visually similar, note that the bin size of P1’s histograms are 50% greater than those of P2 so P1’s moves are clearly faster. The shapes of P2’s two distributions are similar, whereas the positive skew of P1’s mean speed distribution is greater than that of maximum speed. This might be explained by P1 normally using two hands and P2 one, which is consistent with the distributions of numbers of simultaneous digits in use (Fig. 11); obviously, a single touch is one hand, whereas three or more touches are likely to involve two hands. Furthermore, patterns are obvious from the visualizations and from watching the videos. For example, P2’s trails in Fig. 3 between 1:28 and 1:34 show single-hand use, with occasional stationary touches of the other hand. P1’s two-handed interaction is clear from the two sets of multiple coordinate touches in Fig. 2, 0:52–0:58. This serves to demonstrate how MIDAS-analyser not only satisfies many of the data-coding and measurement requirements (Phase 2), including R2.1, R2.2, R2.3, R2.4, and R2.5, but shows how together the different aspects of the data provide comprehensive sources of triangulated evidence to formulate rich explanations of tactile behaviors.

Beyond overall participant performance characteristics, the digit-position data from MIDAS-analyser can be used to investigate each participant’s interaction strategies. Areas of interest in the stimulus can be defined in a similar manner to how they are defined in eye movement research. In the case of the shape stimulus, let us simply consider all nine shapes by splitting the stimulus area into a uniform 3×3 grid. The nine areas are then used to associate each touch with a shape so counts or durations of touches for each shape can be computed.

**Fig. 13:**
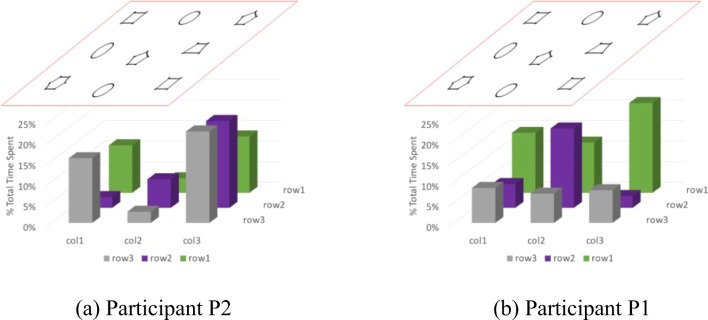
Proportions of time spent in each area of interest by the participants

Figure 13 shows the proportions of time that the two participants spent in the areas associated with each object. The positions of the bars in the graph match the locations of the objects in Fig. 1. Examining the two figures, we observe that P2 spends more time on the five polygons than on the ellipses, which might suggest that the polygons are more challenging for her to perceive. For P1, the long durations are observed for the top row, which might suggest that that row is being used strategically as a datum from which to coordinate the search of the rest of the stimulus. Given the nature of the task, which involves both search and recognition activities, these interpretations are likely to be too crude, but they serve as illustrations of the types of analysis that may be conducted with the data from MIDAS.

The interpretation of Fig. 13 also highlights the need for methods that go beyond aggregated data in order to investigate the underlying processes and strategies of complex tactile interactions. We introduce MIDAS-TPA as such a method.

### MIDAS-TPA

MIDAS-TPA is a method to probe the complexities of tactile interaction strategies. TPA is a manual method conducted by human analysts with the support of dedicated tools. MIDAS-TPA is presented using examples from our shape-matching task.

The challenges for MIDAS-TPA are substantial for many reasons—in particular: coding of data spanning broad spatial and temporal scales (R2.1); coverage of diverse behavior patterns (R2.3); coding of the parallel streams of multi-touch data (R2.4); and coding from separate and integrated perspectives of finger/hands and stimulus (R3.1–R3.4). Furthermore, unlike coding schemes for verbalizations, where words serve as well-defined elementary units of meaning, in TPA there are no such independently meaningful units. Elementary TPA actions can be defined, but these are akin to letters rather than words.

The following subsections introduce the TPA coding scheme for elementary actions and its four-step coding method. Insights obtained from the MIDAS-TPA for the shape-matching task are presented afterwards.

#### Action coding scheme

In MIDAS-TPA, a code for each elementary action or gesture has this format:
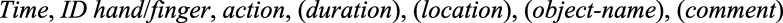


*Time* is the time when the start of gesture is observed. The *ID hand* is recorded as the left or right hand (LH or RH, respectively). *Finger* is recorded as R*N*(*M*), with *N* indicating which fingers of the RH are touching the named object, and *M* indicating the fingers that are touching the display but not the object; “T” and the digits 1 to 4 are the thumb, index, middle, ring, and little fingers, respectively. The same format applies to the LH fingers: L*N*(*M*). (In MIDAS-analyser, figure and hand IDs can be obtained from the video.) The ID of the *action* or list of *actions* observed is described in Table 1. Note that some actions are within an object, whereas others occur between objects. The *duration* and *location* of an action are how long the action lasts and the position(s) of the action. Locations are specified in terms of a coordinate system that is appropriate to the stimulus and task. For the search task, we used the 3×3 rectangular grid that defined the areas of interest, where the top left is (1, 1) and the bottom right is (3, 3). *Object-name* is a label for the object on which the action is conducted. A *comment* may be attached to the code to provide additional details, as required. The items in parentheses are optional, because some actions may be momentary, some may not be associated with any specific location or object, and some may not need commenting upon.

**Table 1:** Description of action labels for TPA. Time scales are short = s (<< 0.5 s), medium = m (≥ 0.5 s & ≤ 2 s), or long = l (>> 2 s)

	Action	Time Scale	Definition
Actions within an object	Trace	s, m, l	Touch on part or whole of a line or perimeter of the object. Single direction of motion, no backtrack.
	Glance	s	Touch a point of an object without stopping, but perhaps slowing.
	Fix	m, l	Hold stationary touching surface. The surface could be a moving touch in before, after, or both, for this action.
	Brush	m, l	Back and forth (≥ 2 traces/glances in opposite directions) touches continuously on part or whole of a line or object. Varying direction of motion.
	Scan-Within	s	One movement in any direction while touching the surface.
	Tap	s	Momentary point touch(es), but no horizontal movement. A lift off of the digit(s) and return to the same location.
	Hover	m, l	A prolonged lift off of the hand or digit(s) and return to the same object, with no intervening translation of finger.
Action across objects	Scan	m, l	Move across a distinct sequence of objects in order; e.g., row, column. A scan follows object order and may pause at intermediate objects.
	Comb	m, l	Set of successive actions occurring in a meaningful area—for example, 3×2 adjacent objects, following the axis. Systematic actions performed at some spatially organized objects. It may consist of multiple scans.
	Skim	s, m	Movement in contact with surface without single definite end object or location. It does not follow a particular sequence of objects. It does not pause at particular objects. It may involve palms.
	SlideTo	s	Move of digit(s) between a start and end object/location. There is contact with the surface throughout. It does not pause at any intermediate objects. It is for the coder to decide whether to code “glances.”
	JumpTo	s	Move of digit(s) between specific start and end object/location, breaking contact with surface.
Actions across and within objects	Span	s	Stretch digits of one hand between original and new location keeping some contact with original location.
Actions above or away from the display	Park	m, l	Hand stationary directly above the reading surface, not on the surface.
	Break	m, l	Hand moves away from the surface.

To identify elementary actions, we reviewed video and MIDAS logs for a broad range of tasks. Thirteen actions were found that are classified in terms of their relation to tactile objects, and may be at an object, across multiple objects, both of these, or none of them. The actions are described in Table 1, and a demo video has been produced to show actual examples of each action (https://bit.ly/2xFFEDn). No claim is made that all possible elementary actions have been identified.

#### Coding method

To cope with the complexities of coding tactile interactions, we adopted a systematic approach consisting of four stages: (1) *specification* of analysis levels and stimuli structure; (2) *first-pass coding* of a trial into main sequences of events; (3) *detailed coding* of selected sub-tasks or sequences within the trial; and (4) *quantification of behaviors* using measures that aggregate the lines of code in various ways. The splitting of coding into first-pass and detailed coding, Stages 2 and 3, is a pragmatic way to manage the complexity of coding over broad spatial and temporal scales (R2.1). For the coding activities we exploit the functionality of a spreadsheet to organize the disparate information within the codes and across multiple nested levels of codes.

**Fig. 14:**
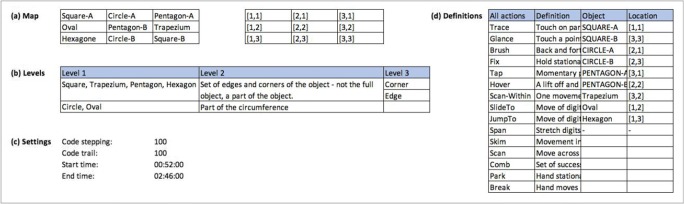
Example of the spreadsheet tables used for the specification step in the coding method

*Specification* lays the foundation for the analysis by setting the overall parameters of the analyses. (1a) A positional reference system for task relevant features or objects in the stimulus is defined. For the shape-matching task (Fig. 1), the objects are given unique names and their locations given by the 3×3 grid—Fig. 14a is a table from the spreadsheet that defines the shape names and locations. For more complex tasks and stimuli, hierarchical feature-naming and coordinate schemes may be appropriate. (1b) The target of tactile actions may focus on different sections (levels) of stimuli, so stimulus levels are defined at different levels of granularity and depend on the types of interaction that are of theoretical interest (R2.1). For our shape-matching task, we defined a top *Level 1* as actions on whole individual shapes (e.g., hexagon), an intermediate *Level 2* for actions on some features of a shape (e.g., top half of the hexagon only), and a bottom *Level 3* for actions on individual sides and corners of shapes (e.g., top edge of the hexagon only). These levels are identified in the spreadsheet table in Fig. 14b. In general, the number and grain size at each level will depend on the types of phenomena of interest. (1c) Given the levels set in (1b), the settings of MIDAS-analyser “stepping” and “trail” values can be specified, so that replays reveal touch patterns at an appropriate level of detail. For example, for the shape-matching task, 100-ms stepping values were initially chosen to decompose P2’s traces around the perimeter of objects (Fig. 14c).

For ease of coding, all the TPA action codes, the object names, and their locations are listed in the spreadsheet (Fig. 14d), so that they can be referred to by the spreadsheet’s built-in functions that automatically generate pull-down lists for entering cell values (see below). To distinguish objects that are target pairs in the shape-matching task, their names are entirely capitalized, whereas the single distractor items are in upper and lower case (column “Object” in Fig. 14d).

**Fig. 15:**
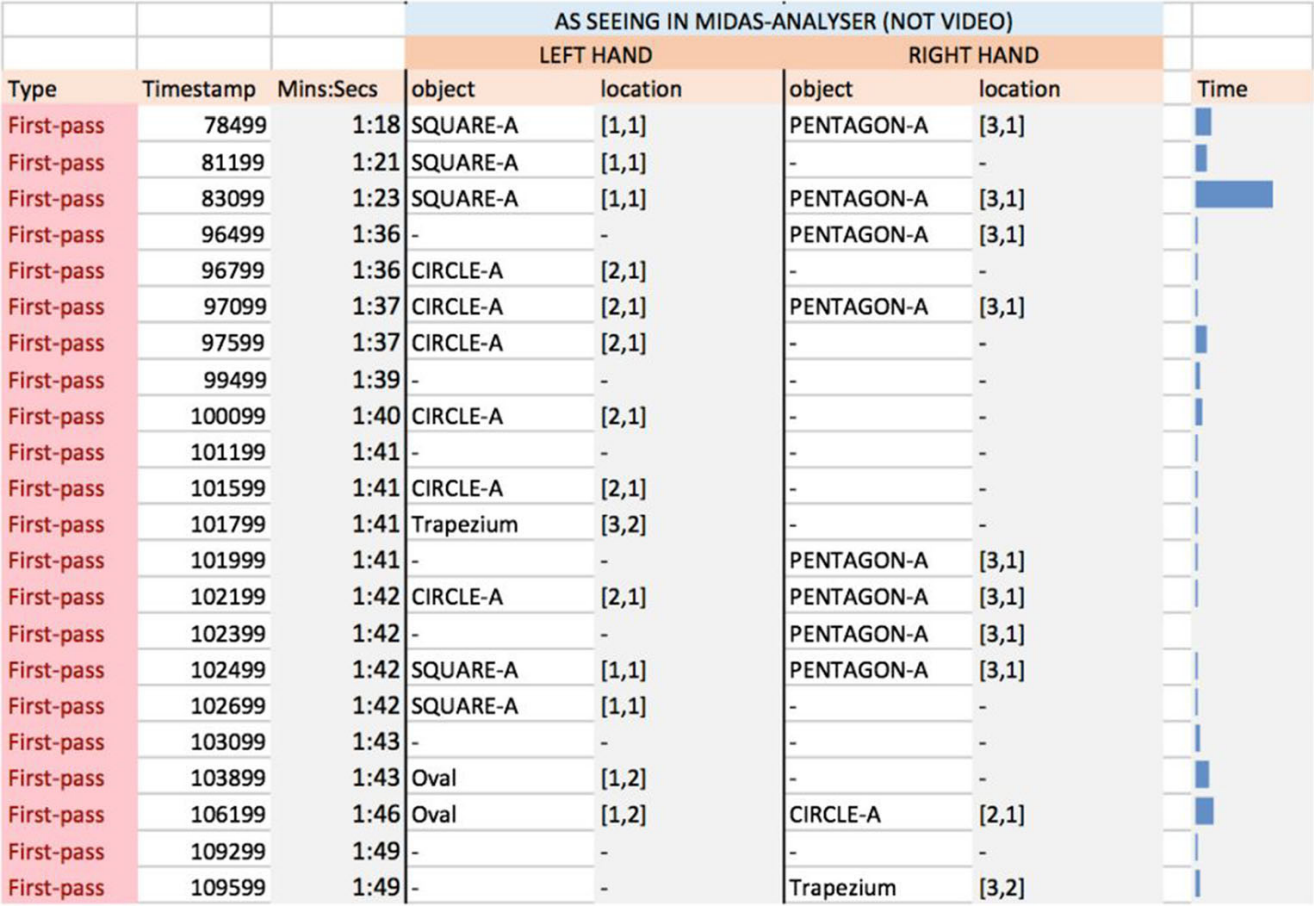
Snapshot of the first-pass coding from 1:18 to 1:49 for Participant P2

The purpose of a *first-pass coding* is to generate an initial segmentation of the task, to reveal the overall pattern of behaviors, and thus to identify subtasks that are likely targets for coding at the most detailed levels. Figure 15 shows a section of the first-pass coding for P2, spanning 31 s. The “Timestamp” is the time (in milliseconds) obtained from reading the MIDAS-analyser “current time” in the control panel (Fig. 10); the column “Mins:Secs” is time in a human-friendly format computed by the spreadsheet. The middle columns of the table give the names and locations of the objects being touched by each hand; these are filled out manually using MIDAS-analyser. Each line codes the ongoing actions in particular areas of interest. For example, at time 1:18, the left hand is reading SQUARE-A, at location [1, 1], and the right hand is reading PENTAGON-A, at location [3, 1]. New lines are generated whenever actions begin or cease by one or both hands. For example, when the right hand is briefly raised from PENTAGON-A we have a new line at 1:21 just for the left hand, and at 1:49 a new line begins, because both hands are briefly lifted from the stimulus. To code an action that runs over more than one location, a range of locations may be added on that line.

The “Time” column on the right of Fig. 15 uses the built-in functionality of the spreadsheet to graphically show the duration of each first-pass action: This is relatively uneven, with P2 spending a long time comparing a square and a pentagon simultaneously, for instance. P2’s right hand remains on one of the pentagons for most of the time, whereas the left hand makes returns to previously read objects.

**Fig. 16:**
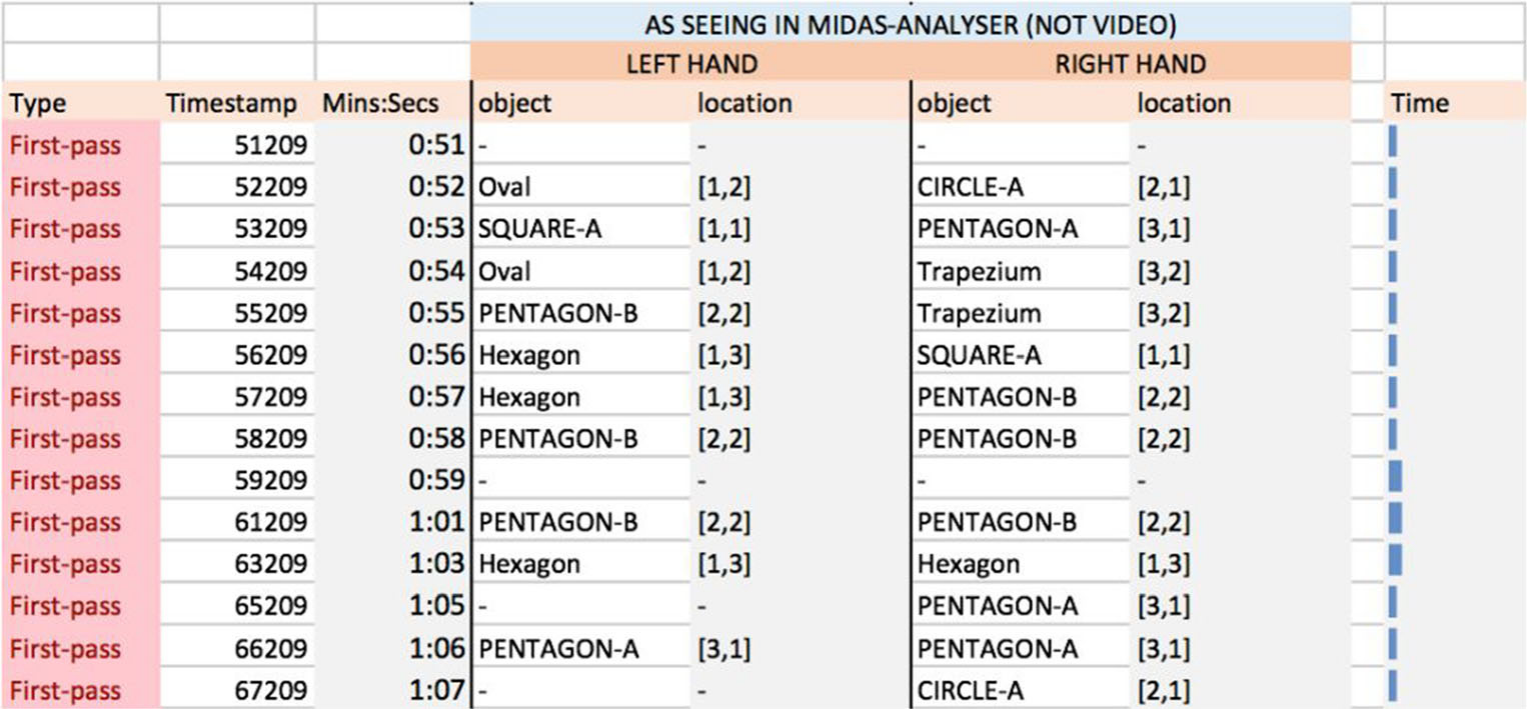
Snapshot of the first-pass coding from 0:51 to 1:07 for Participant P1

Figure 16 shows a section of the first-pass coding for P1 for a period spanning 15 s. The time spent reviewing objects is relatively constant across the different events. Unlike for P2, both hands are typically in action and touch a variety of objects, with some objects being touched successively by the two hands (e.g., PENTAGON-B at 0:58).

From the first-pass codings presented in Figs. 15 and 16, we observe that P1 had touched several shapes within a few seconds of starting the task, whereas P2 moved across only two shapes during the same time frame. Furthermore, P1 explored objects across all three rows in that time, suggesting that right from the outset P1’s strategy was to gain an overview of the distribution of objects, whereas P2 seemed to compare or identify objects as they were found in the stimulus area. Thus, the first-pass coding not only allows us to keep a record of the overall sequence of actions, but it provides an overview of the potential strategies that participants seem to be taking when solving the task. This goes some way to MIDAS-TPA fulfilling requirements R2.1 and R2.3, which consider the range and diversity of behavioral patterns. The next stage contributes further.

**Fig. 17:**
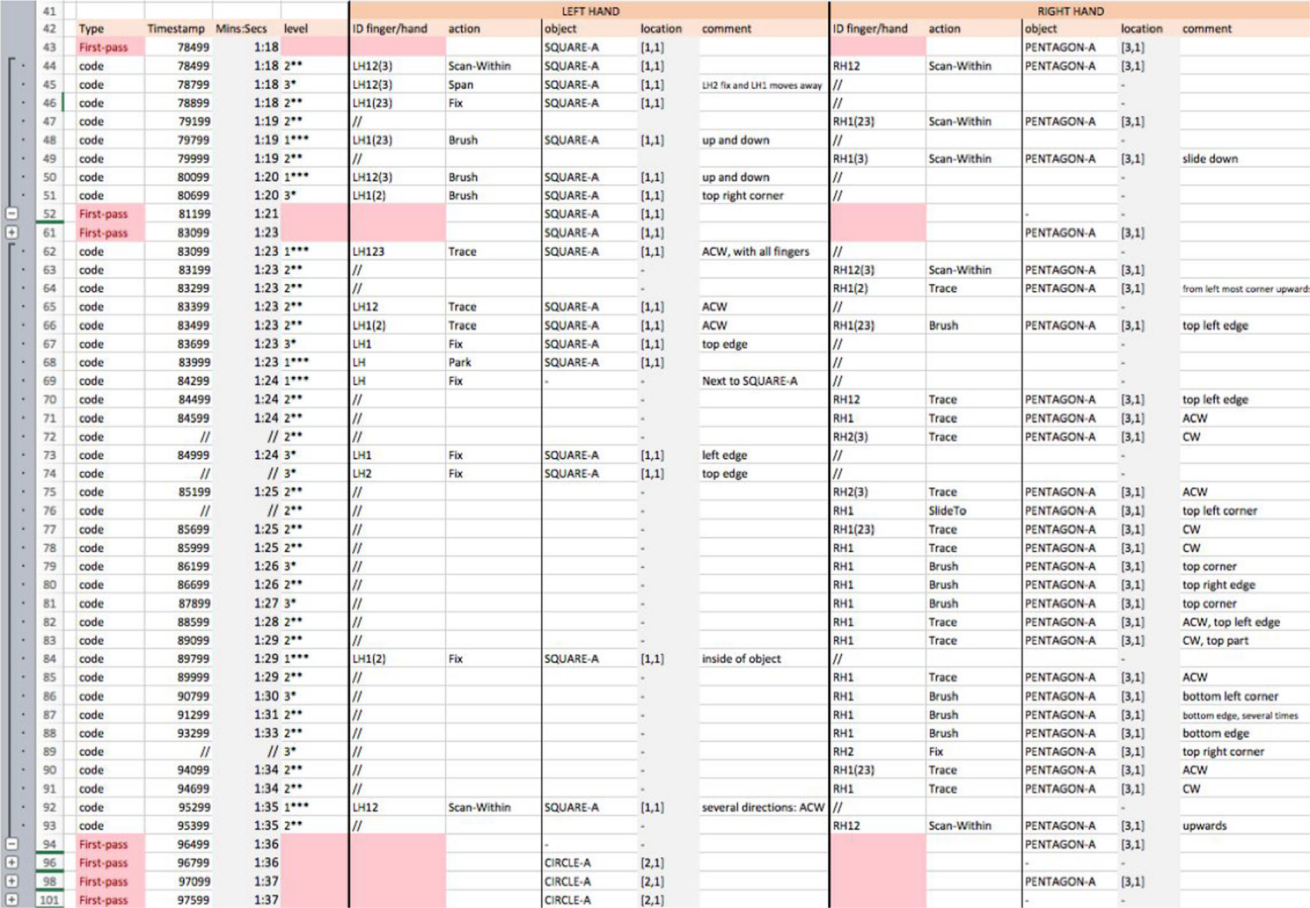
Snapshot of the detailed coding for Participant P2—both hands

*Detailed coding* follows a first-pass coding. Figure 17 shows a section of the detailed coding for P2, which progresses by selecting a line of the first-pass coding and then adding new lines with details of actions at a finer granularity under it. Lines added during the first-pass coding are in red and lines added during the detailed coding have a white background only (see the “Type” column in Fig. 17). The spreadsheet’s built-in functionality for hierarchically defining and selectively displaying groups of lines has been used to manage display of the protocol (Fig. 17, far left, plus and minus signs). In Fig. 17, just the detailed coding for the segments between 1:18 to 1:20 and 1:23 to 1:35 are shown; the rest of the data are hidden. The shown data are bracketed (left) and indicated by the red cells at the first-pass level. The spreadsheet also includes extra columns for completing the full specification of each elementary action, which include details of the finger identity, gestures, and comments. In the “level” column, the level of the gesture is specified; for example, at line 66 this is Level 2, since the hand is tracing part of the square, but in line 67 the level is 3, since the left-hand index finger is fixed on an edge of the square only. Due to the amount of data presented in the spreadsheet, the digit for the level has been augmented with a visual cue to aid perception of the levels.

P2 reviews a square with the left hand for an extended time from 1:18 to 1:35. The column “action” shows the gestures recorded during this time. The symbol “//” indicates that “the gesture continues as above.” For example, there is a “fix” gesture on the top edge of a square at 1:24 (line 74) with LH2, and this persists for a few seconds, until the participant changes the digits used at 1:29 (line 84).

**Fig. 18:**
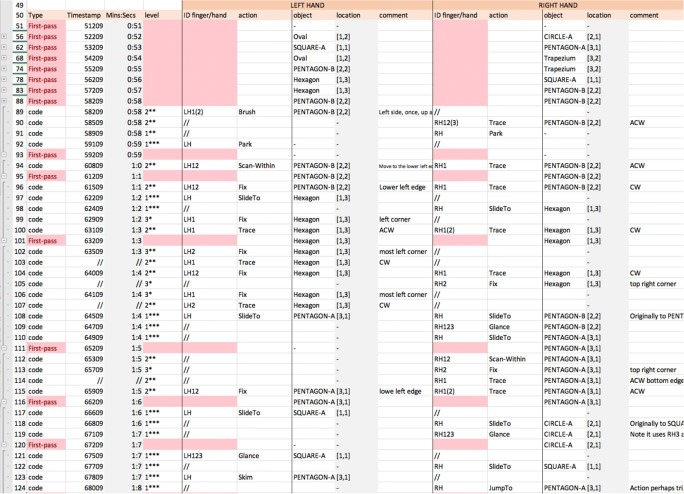
Snapshot of the first pass coding for Participant P1—both hands

Figure 18 shows the codings for both hands for P1. It can be appreciated that gestures (actions) differ from one hand to the other. As in Fig. 17, Fig. 18 shows segments of detailed coding with a white background (see the rows under the “Type” column), whereas rows in red are the first-pass coding rows.

P1 moves more quickly across shapes than does P2 and shows a more varied set of gestures too. A quick look at P2’s detailed coding (Fig. 17) reveals that the most frequent gestures seem to be “trace,” “brush,” “fix,” and “scan-within”; however, P1 also shows gestures such as “slideTo,” “glance,” “skim,” and “jumpTo.” A key difference between these sets of behaviors is that P2’s gestures are aimed at reviewing or identifying a shape, whereas P1’s gestures include both gestures that indicate indexing of objects (e.g., fix, slideTo, jumpTo) and gestures that indicate recognition of objects (e.g., brush, trace).

A detailed analysis of the protocol segments has been provided so that readers can judge how MIDAS-TPA provides data to address many of the requirements. In particular, multitouch coding (R2.4, R1.2) and diverse behavior coding (R2.3) are integrated across broad spatial and temporal scales (R2.1).

#### Examples of interesting behaviors observed from the detailed coding

To give a sense of the richness of tactile behaviors and strategies that may be discovered using MIDAS-TPA, we outline some differences between P1 and P2 that we have found.

Figure 18 presents some of the detailed coding for P1. As can be observed, both hands are used to solve the task; there is no dominant hand in that respect. However, some gestures seem to be conducted with one hand more often than with the other. An example of this is “trace”: P1 seems to use the right hand to trace objects slightly more often than the left hand. A fuller review of this is in the next section.

**Fig. 19:**
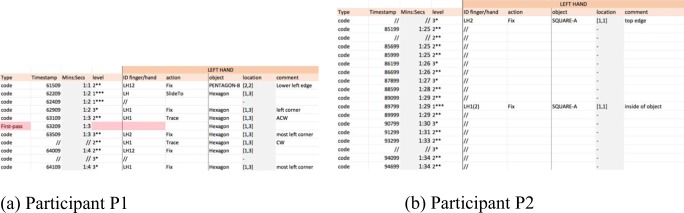
Snapshots of the “fix” gestures from the detailed codings for (a) Participant P1 and (b) Participant P2

Observe how the durations of a gesture differ between participants, with P1 performing gestures in very short time spans. An interesting example is the case of “fix” (Fig. 19). An extract from the detailed coding for P1 shows that the “fix” gestures last one second or less (Fig. 19a), whereas the “fix” gestures for P2 can last several seconds (Fig. 19b). Furthermore, this gesture works as an index to a full object for P2, whereas for P1 it works as an index of an object feature (e.g., a corner), too.

**Fig. 20:**
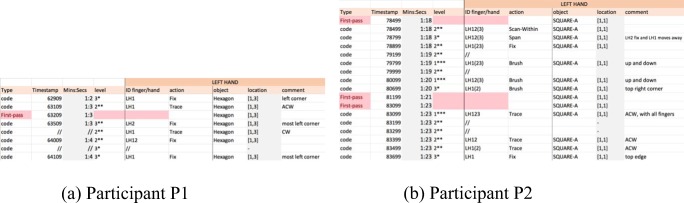
Extract of the Level 3 gestures for (a) Participant P1 and (b) Participant P2

Other differences can be spotted in relation to the granularity (“level”) of the gestures used by both participants. For example, records of Level 3 gestures, which have the lowest granularity, were not only found slightly more frequently in the detailed coding for P2 than for P1, but they were associated with different gestures too. Figure 20a shows an extract of P1’s detailed coding, showing that gestures with Level 3 are “fix” gestures only, whereas P2’s Level 3 gestures (Fig. 20b) are “span,” “brush,” and “fix.” It seems P2 was trying to identify individual parts of the objects (e.g., an edge, a corner), but P1, who has more experience reading tactile materials, takes another approach and may focus on, perhaps, a more abstract view of the objects, fixing on specific features only to use them as reference points.

**Fig. 21:**
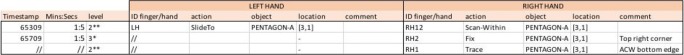
Extract of the code for Participant P1. The right hand performs two different gestures simultaneously

TPA also allowed us to observe simultaneous gestures performed with one hand. Figure 21, shows a three-line snapshot of the detailed coding for P1. It shows that the LH was sliding to Pentagon-A while the RH was already scanning Pentagon-A. However, at 65709, before the LH reached the object, P1 fixed the RH middle finger at the top right corner of the pentagon and traced the object (counterclockwise) with the RH index, simultaneously. Other similar examples of simultaneous gestures seemed more frequent with P1 than with P2, too. In this example, the reader can appreciate the granularity at which the gesture is observed and recorded.

Generally, creating a first-pass coding for our matching task took between 15 and 30 min. However, the detailed coding took considerably longer when the settings specified above were used (Fig. 14c, 100-ms stepping): between 2 and 3 h of detailed coding per minute, per hand. Thus, it is relevant to be selective regarding what parts of the first-pass coding that need to be coded in detail and to specify the coding settings thoughtfully. The first-pass coding and detailed coding of P1 generated just under 200 lines of code, whereas for P2, the coding generated over 600 lines of code.

Although we do not present an example here, the reliability of the coding should be evaluated. Given the complexity of the data, the interrater comparison for tactile protocols will be more challenging than that for simpler, verbal protocols. It is recommended that multiple assessments be conducted on different aspects that underpin findings that will provide evidence for theoretical claims. Coding for the position and timing of touches is relatively unproblematic, given the MIDAS log data, but assigning codes to actions requires more judgment on the part of the analyst. If reliability cannot be achieved at the level of the named actions in Table 1, we have the option to conduct a coarse-grained analysis based on the categorization of actions as within or between objects, and their duration, which might still be sufficient to differentiate strategic differences between groups.

#### Quantifying participants’ gestures: Time spent over the first 41 s

Deriving *quantitative measures* is the final stage of MIDAS-TPA, which aggregates various aspects of the gestures coded. An example with our sample task follows, as an exemplar of how data from a MIDAS-TPA may be quantified, and thus reveal overall differences between participants (or populations) that go beyond the descriptive level of the protocols themselves.

**Fig. 22:**
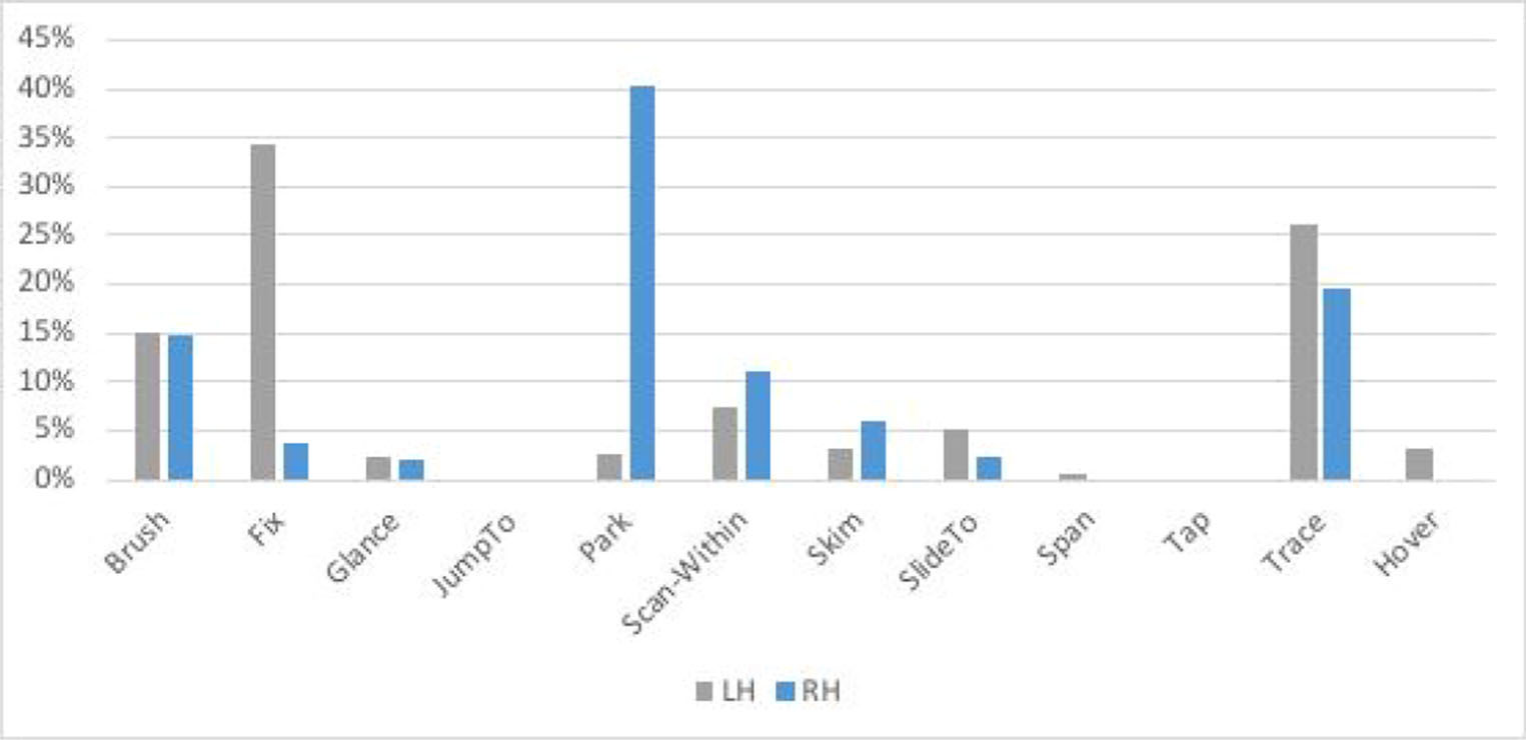
Proportions of time spent on each gesture for Participant P2 during the first 41 s. The graph plots the proportions of time for the left hand (LH) and the right hand (RH)

**Fig. 23:**
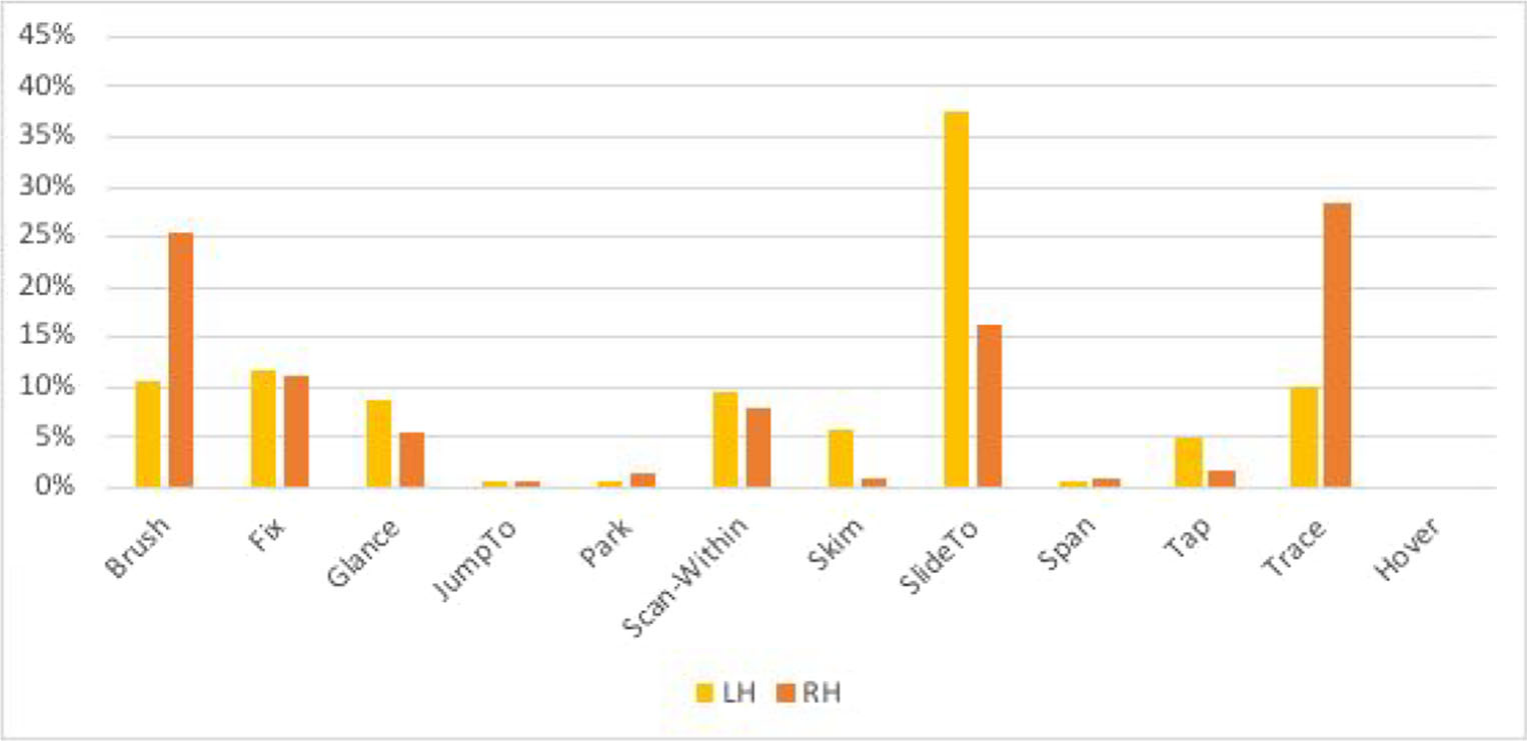
Proportions of time spent on each gesture for Participant P1 during the first 41 s. The graph plots the proportions of time for the left hand (LH) and the right hand (RH)

The graphs in Figs. 22 and 23 plot the time spent on each coded gesture for P2 and P1, respectively, during the first 41 s, which is the length of time that P1 took to finish the task.

In P2’s chart (Fig. 22), large bilateral differences are prominent in the “park” and “fix” gestures, which register the absence of touch interactions. In contrast, the gestures associated with detailed inspection of shapes, “trace” and “brush,” are much more equal, despite the participant being right-handed. This suggests that P2 tended to engage one hand at a time, which is consistent with her use of a small number of digits (Fig. 11). It also suggests that she used each hand equally, and independently, to inspect the shapes closely, while the other hand tended to be at rest. The difference in the mode of resting, right hand off the surface (“park”) and the left hand on (“fix”), appears arbitrary. The traces in frames 1:26 to 1:32 appear to be an example of this behavior. This is consistent with the trace patterns in Fig. 3.

In contrast, the chart in Fig. 23 shows that the right hand of our right-handed P1 dominated in the “brush” and “trace” shape recognition gestures, whereas the left hand dominated “slide to” gestures between shapes. The near-continuous movement of both hands is clear from the small percentage of “fix” and the tiny percentage of “park” gestures, which contrasts with the one-hand-at-a-time performance of P2.

#### MIDAS-TPA results

MIDAS-TPA is a versatile approach to studying and closely analyzing the gestures of tactile graphic readers. All the codes specified in our coding scheme (Table 1) were used in this study. Furthermore, the codes allowed the observation of distinctive behaviors and strategies carried out by both participants. Although we do not claim that the codes cover all possible tactile actions, they were sufficient to express all the gestures used by participants with different tactile reading expertise while performing our shape-matching task.

Both hands can be coded in fine detail, as well as the actions of individual fingers. Furthermore, some codes may allow the matching of behaviors to specific cognitive tasks—and this can be pinpointed in the protocol by using the corresponding timestamp too. For instance, a “fixation” on an object or “jumping to” a previously visited object could be associated with indexing of that object; “trace” and “brush” of an object could be associated with object recognition; or tracing different objects with both hands could be associated with coordination of features for comparison of objects.

The four phases of the coding method proved to be structured and flexible for the analysis of tactile behaviors. The structured process allows the coder to start the analysis by recording and obtaining an overview of the overall tactile behavior first, and then it permits an in-depth selective coding to explore aspects of interest. The method can be used with other complex diagrammatic representations by carefully defining the areas of interest during the specification step. MIDAS-TPA can be used with participants with different expertise too, as was shown in previous sections.

Thus, it has been demonstrated how MIDAS-TPA may be used to quantify and qualify participants’ behaviors. For example, differences between P2 and P1 were reflected in the granularity (level) at which they reviewed objects for the matching task, and in the frequency of conducting several actions simultaneously with both hands. Gestures of participants were quantified and this provided an insight into the main role of each hand, as well as characteristic behaviors of P2 and P1.

## Discussion

In this final section, we consider the potential and limitations of MIDAS in two ways. First, MIDAS will be judged against the set of requirements for each of the three stages of tactile interaction experiments. Second, the implications of preliminary findings about the system demonstration will highlight how novel aspects of tactile interaction may be investigated.

### Assessment of MIDAS against the system requirements

Capturing data is the first stage of tactile interaction experiments. MIDAS-logger satisfies most of the requirements. Because it exploits current touch screen technology, good spatial and temporal accuracy and resolution are achieved at a level that meets the first requirement of cognitively oriented tactile interaction studies (R1.1). This technology also underpins the system’s ability to capture data for up to ten fingers (R1.2) and the automatic digital recording of position and timing data for touches (R1.4). However, tablets have no capability to determine the identity of the digits in contact with the touch surface (R1.3) and some tablets may occasionally suffer from an input overload. Although participants can be easily trained to cease touching briefly in response to an overload signal, this disturbs the flow of the task. Fortunately, on certain models of tablets this problem is rare (see above). MIDAS-logger does require an initial calibration procedure in order to record the stimulus position for MIDAS-analyser to align the screen and stimulus coordinate systems, but otherwise no further calibration is required either during the running of a trial or during analysis (R1.4). Data about off-surface movements of fingers and hands is not captured (R1.5), nor is information about the force of touches. The approach is not invasive, even in a minimal form (R1.6). MIDAS-logger meets the main practical requirements (R1.7). Its use of everyday technology means that it is economical. It is compatible with common forms of tactile materials, such as swell paper, plastic embossing (“German”) film, and thermoform stimuli, with the provision that features are not so wide and tall that the finger pad is completely elevated from the surface. Although the wooden frame speeds up the switching of stimuli, its main purpose is to mask the small area at the periphery of the screen that activates the generic tablet menus. In summary, the MIDAS-logger satisfies more of the data capture requirements than the previous systems described above.

The second stage of tactile experiments is the coding and derivation of measures. The requirements of this stage relate both to MIDAS-analyser and to MIDAS-TPA. The interactive visualization of touch traces in MIDAS-analyser supports the analyst’s examination of tactile behaviors over broad spatial and temporal scales (R2.1) through flexible selection of diverse playback display parameters, and even direct instantaneous manual control of the playback and rewind at whatever the speed an analyst wishes. MIDAS-analyser’s output of structured data allows investigators to use standard data analysis packages to filter, aggregate and test the touch data at will. A specific aim in the development of MIDAS-TPA was to support the detailed coding of touch actions across broad spatial and temporal scales (R2.1), which is realized as flexible temporal and spatial code schemes. Obviously, diverse measures of behaviors can be derived from the structured data (R2.2), but MIDAS-TPA in addition enables tactile interaction strategies to be identified from the rich patterns of the meaningful action codes (R2.3). MIDAS-analyser and MIDAS-TPA both support multi-touch coding (R2.4). It is particularly worth noting that the integration of the video data with the touch trace data enables the analysis of actions in relation to specific hands and individual fingers, which compensates for the absence of finger identity in the raw touch data (R2.5). The combination of the touch and video data provides sufficient information to identify subtle behaviors, such as when a finger follows the outline of a shape whilst accompanying other digits but does not itself touch the shape, or when there is a brief slowing of the fingers as they “glance” at a shape, perhaps indicating that the participant recognized the shape even though the overt action just appears to be a “skim.” Although automatic coding of behavior patterns and the computation of measures (R2.6) would be desirable, such features are yet to be developed within MIDAS. As compared to the previous approaches described above, MIDAS-analyser and MIDAS-TPA appear to satisfy more of the data coding and measure derivation requirements.

The last stage of tactile interaction experiments encompasses interpretation and analysis activities, which may take three different perspectives: a finger or hand focus (R3.1), a stimulus focus (R3.2), and both combined (R3.3). MIDAS-analyser and MIDAS-TPA support interpretations separately about participants’ use of fingers and hands (e.g., Figs. 11 and 12), about the locations and durations of touches associated with particular objects in the stimuli (e.g., Fig. 13), and about all of these combined (e.g., Figs. 17, 18, 19, 20, 21, 22 and 23). Combining both perspectives used in the analysis of braille reading, MIDAS goes further by extending the state of the art to the complexity of strategies for full 2-D stimuli. Unlike Breidegard (2007), we have not yet attempted to integrate touch analysis with participants’ verbalizations, although it is quite feasible to augment MIDAS-TPA with transcriptions of utterances that participants make concurrently with their touch interactions (R3.4).

In summary, the three parts of MIDAS satisfy most of the desirable requirements of an approach to the study of tactile interactions, which were derived from considering the capabilities and limitations of existing methods in the literature.

### System demonstration

The comparison of the performance of our two selected tactile graphic readers primarily served to illustrate the capabilities of the MIDAS system. However, as we believe that this is the first detailed systematic analysis of the differences between participants with and without experience in reading tactile graphics, certain implications should be highlighted to guide future work in this area, even though we make no claims about the representativeness of each participant’s performance to some wider sub-population.

The most striking observation is that readers of tactile graphics cannot be treated as a homogeneous group. Many substantial differences in performance manifested between our two participants, just on the simple shape-matching task presented here. Participant P1 (P1) was not only substantially quicker and more accurate on the task, but differences were apparent in other performance measures and in terms of higher level strategic behaviors. One key difference is P1’s constant use of both hands and, at some points, each hand was seemingly performing a different function, such as marking a location versus perceiving a shape. This resonates with the research by Lorimer (2002), who suggests that the most efficient method for reading braille is when hands work independently and take different roles (e.g., one hand reads, the other is positioned elsewhere along the line). Furthermore, Lorimer states that a poor reader would tend to use both hands for the same purpose (p. 77), which parallels Participant P2’s use of both hands to recognize an object more often than P1.

P1 used more fingers more of the time than P2, but this cannot merely be attributed to the former’s deployment of two hands, as she displayed sophisticated behaviors with just one hand, such as marking (remembering) a location with the thumb whilst feeling a shape with the other digits of the same hand. The speed of movements of the hands over the tactile graphic was substantially faster for P1 than for P2. In part this may be explained by P2 focusing laboriously on an object’s details (e.g., a corner, an edge), often in order to work out its shape. In contrast, P1 was able to recognize the shape of an object even during a *skim* over the object, without any overt sign of having attended to its form. We are certain of this interpretation, because P1 correctly declared that the bottom right square in the stimulus matched the top left square after only having touched the square once earlier in the trial in a continuous skim action. Although P2 seemed to construct the shape of objects by deliberately integrating features of objects across successive *trace*, *brush* or *scan-within* actions, P1 appears to be able to perceive shapes directly in the form of dynamic patterns under her moving fingers. This raises the issue of whether variations in the strategies of tactile graphic readers might reflect differences in the strategies of proficient and poor readers of braille. Proficient braillists make continuous sweeps across multiple cells when perceiving individual words, rather than recognizing words by assembling them letter by letter.

Comparison of our participants reveals a rich range of interesting but unexplored behaviors and strategies for interacting with tactile graphics and interfaces. We have illustrated the potential of MIDAS-logger, MIDAS-analyser and MIDAS-TPA as a set of tools for studying such phenomena.

### Applications of MIDAS

To conclude this article, we briefly consider the types of studies that MIDAS now makes feasible. Our sample experiment contrasts individual differences between users with low and high levels of experience in the use of tactile graphics. Obviously, we can study how users with the same level of experience fare with alternative graphics, for example to test the efficacy of alternative designs of tables, graphs, and charts. MIDAS permits such studies to probe beneath the overall efficacy of particular formats of representations (based on speed and accuracy measures) and allows the evaluation of specific design features within representations by exploiting high spatial and temporal resolution data provided by MIDAS.

The many studies of braille reading phenomena cited in the introduction could be repeated, but now fully taking into account the role of multiple simultaneous touches. Additionally, the richness of MIDAS data could be used to derive measures of braille competence that are more nuanced than whole task performance measures. For example, what are the relative merits of different micro-reading strategies that might occur below the level of regressions to earlier words in a line and returns to the beginning of new lines (cf. Millar, 2003)?

Of course, MIDAS will function without a tactile stimulus pinned to the surface of the tablet, so the system may be used as a generic approach to the study of multitouch interaction on touch screens. Furthermore, and in contrast to current modes of interaction on mobile device touch screens that are limited to two digits (e.g., pinches), our observations, such as those in Figs. 2 and 3, provide evidence that humans are capable of far more sophisticated multitouch gestures, in which digits on the same hand might even perform different operations, for instance. We shall use MIDAS to study such interactions.
